# Mechanonociceptive tactile feedback enhances prosthetic grasp control during repeated human-in-the-loop interaction

**DOI:** 10.3389/fnbot.2026.1910158

**Published:** 2026-10-15

**Authors:** Elif Hocaoglu

**Affiliations:** 1 Living Robotics Laboratory, Department of Electrical and Electronics Engineering, Istanbul Medipol University, Istanbul, Türkiye; 2 Living Robotics Laboratory, Department of Biomedical Engineering, Istanbul Medipol University, Istanbul, Türkiye; 3 Research Institute for Health Sciences and Technologies (SABITA), Istanbul, Türkiye

**Keywords:** grasp control, human–machine interaction, innocuous painful feedback, mechanonociceptive feedback, neurorobotics, sensory feedback, tactile feedback, upper-limb prosthetics control

## Abstract

Upper-limb prosthetic systems continue to face a fundamental challenge in providing sensory feedback that remains functionally effective during repeated interaction. This study investigates innocuous painful feedback (IPF), a controlled mechanonociceptive tactile modality intended to provide salient error-related information during human-in-the-loop prosthetic interaction. A custom experimental platform was developed to emulate prosthetic grasping conditions in healthy participants, enabling systematic evaluation of IPF across feedback modality, stimulation frequency, temporal modulation strategy, and spatial configuration. IPF was associated with lower grasp-position errors than visual-only and visual-plus-force feedback, supporting its potential as a corrective sensory feedback modality for boundary-constrained grasp control. Within the tested frequency range, high-frequency stimulation produced the lowest error levels, demonstrating that the functional effectiveness of IPF depends on stimulation frequency. Progressive frequency modulation generally maintained lower error levels than fixed-frequency stimulation across repeated interaction, while sequential dual-site stimulation yielded lower overall participant-level errors than repeated single-site stimulation, although the magnitude of this difference varied across the trial sequence. Collectively, these findings indicate that the behavioral effectiveness of mechanonociceptive feedback depends not only on feedback modality, but also on its temporal and spatial organization. As a proof-of-concept study conducted in neurologically intact participants using a virtual prosthetic-like grasping paradigm, these findings provide a basis for subsequent validation with individuals with upper-limb amputation and physical prosthetic devices.

## 1 Introduction

The human hand achieves dexterous interaction with the environment through the continuous integration of somatosensory, proprioceptive, and nociceptive feedback (Fifer et al., 2022) and neuromuscular control mechanisms. This complex interplay underlies the development of fine motor skills and facilitates adaptability across varying functional contexts. However, individuals who experience upper limb loss or severe hand injuries often suffer from a substantial reduction in functional ability, impairing their performance of everyday activities. To compensate for this loss, many individuals utilize myoelectric prosthetic devices, which are designed to restore partial motor function. These technologically advanced prosthetics play a critical role in enabling users to perform essential tasks such as grasping and manipulating objects, thereby contributing to improved autonomy and quality of life (TASKA Prosthetics, 2024).

Over the past decades, the prosthetics industry has introduced a variety of advanced devices that mimic both the appearance and fundamental functions of the human hand (Össur, 2026; Mobius Bionics, 2026; Steeper Group, 2026; Vincent Systems, 2026). While these innovations have significantly improved the mechanical capabilities of prosthetic limbs (Hocaoglu and Patoglu, 2022a,b), they remain limited by the absence of integrated sensory feedback, an essential component for achieving precise and intuitive control. The difficulty of manipulating small objects, such as coins, while wearing thick gloves illustrates how diminished tactile input severely impairs task performance (Hocaoglu, 2019, 2023). Similarly, individuals with upper extremity amputations, who are deprived of natural somatosensory input and must rely predominantly on visual feedback, often face challenges in executing accurate and coordinated movements with their prostheses. This increased reliance on visual monitoring elevates cognitive load and reduces interaction stability, underscoring the need for effective sensory feedback in prosthetic systems.

Beyond facilitating motor precision, sensory feedback plays an important role in fostering prosthetic embodiment, defined as the user's perception of the device as an integral part of the body. Mechanotactile feedback has been shown to enhance feelings of ownership and agency during active prosthetic control, increase user confidence, and reduce reliance on visual cues (Risso and Valle, 2022; Shehata et al., 2020; Wendelken et al., 2017). Sensory feedback may also have therapeutic relevance for phantom limb pain (PLP). Following amputation, the loss of natural afferent input alters the normal coupling between motor commands and their expected somatosensory consequences, while peripheral and central neuroplastic changes and altered sensorimotor processing have been implicated in the development and persistence of PLP (Ortiz-Catalan, 2018). Restoring behaviorally meaningful sensory information through the prosthesis may therefore help re-establish sensorimotor coupling and modulate pain-related neural processing. Consistent with this rationale, prosthesis-mediated somatosensory feedback has been associated with reductions in PLP while simultaneously improving prosthetic functionality and embodiment (Dietrich et al., 2012; Page et al., 2018), and more recent evidence has demonstrated that direct stimulation of residual peripheral sensory pathways can evoke tactile and proprioceptive sensations while suppressing PLP in individuals with transradial amputation (Soghoyan et al., 2023). Thus, sensory restoration may contribute not only to improved prosthetic control and embodiment but also to the management of phantom limb pain, further motivating the development of sensory feedback systems that are both functionally effective and perceptually stable during prolonged prosthetic interaction.

Extensive research over decades has explored various approaches to address the lack of sensory feedback in prosthetics, including sensory substitution, modality-matched feedback, direct neural stimulation, and targeted muscle reinnervation (Simon et al., 2023; Al-Ajam et al., 2022). Direct neural stimulation involves patterned electrical signals to stimulate sensory afferents via implanted electrodes, while targeted muscle reinnervation transfers transected peripheral nerves to reinnervate intact muscles and skin (Bates et al., 2020). Sensory substitution uses one sensory modality to replace another, with vibrotactile and electrotactile stimuli converting forces sensed on the prosthesis into vibrations or electrical signals delivered to the patient's skin. Modality-matched feedback employs the same modality for sensing and stimulation, relaying forces sensed by the prosthesis directly to the user. Among these, vibrotactile, electrotactile, and force feedback have received particular attention as non-invasive modalities. Vibrotactile feedback employs vibrations to convey tactile information, whereas electrotactile feedback delivers electrical stimuli to stimulate sensory receptors. Force feedback uses mechanical resistance to simulate contact sensations. While each method has shown potential, significant challenges remain, particularly rapid habituation, cognitive overload, and inconsistent user adaptation (Antfolk et al., 2013; del Valle and Navarro, 2013; Nghiem et al., 2015).

Recent advances in non-invasive sensory restoration have further expanded the capabilities of prosthetic feedback systems through somatotopic and modality-matched strategies, which aim to preserve the anatomical correspondence or the physical modality of natural tactile perception. These approaches have demonstrated improvements in embodiment, proprioception, object manipulation, and sensory interpretation (Paskett et al., 2019), while somatotopically congruent feedback has also been shown to facilitate the learning and interpretation of prosthetic hand postures and to support the generalization of artificial sensory information to novel postures (Segil et al., 2020). More recent studies have further demonstrated improvements in haptic perception and proprioceptive performance using modality-matched and hybrid sensory feedback strategies (Sariyildiz et al., 2023), while comparative investigations have suggested that although somatotopic interfaces provide important advantages, stimulation encoding strategies still require further optimization to maximize perceptual performance and usability (Su et al., 2022). Recent review articles likewise conclude that, despite substantial progress toward clinically viable somatosensory neuroprostheses, maintaining perceptually robust and clinically practical sensory feedback during prolonged interaction remains an important open challenge, and advanced stimulation paradigms continue to be actively investigated (Fisher et al., 2023; Graczyk et al., 2024).

Among these emerging stimulation paradigms, nociceptive sensory feedback has attracted increasing attention as a means of restoring protective sensations in prosthetic systems. A neuromorphic multilayered electronic dermis (e-dermis) combined with transcutaneous electrical nerve stimulation (TENS) has been developed to provide amputees with a continuous perceptual spectrum from innocuous to painful touch, enabling object sharpness discrimination and pain-reflex-based protective responses (Osborn et al., 2018). A hybrid sensory feedback framework integrating TENS-based thermal nociceptive warning with a bionic withdrawal reflex has subsequently been proposed to facilitate both voluntary and reflexive release of hazardous hot objects while protecting the prosthetic device from thermal damage (Xie et al., 2024). These studies demonstrate the potential of nociceptive feedback for sensory restoration and protective reflexes. However, unlike these approaches, which primarily employ electrically evoked nociceptive sensations for protective perception and withdrawal responses, the use of mechanically delivered innocuous mechanonociceptive stimulation as an error-dependent feedback modality for prosthetic grasp control has not been systematically investigated. Furthermore, unlike TENS-based approaches that transform mechanically induced grasping events into electrically evoked nociceptive sensations, the proposed approach preserves the physical stimulus modality throughout the sensing-feedback pathway by delivering mechanically induced cutaneous stimulation. Accordingly, rather than replacing recent somatotopic or modality-matched feedback strategies, the present study investigates whether mechanonociceptive innocuous painful feedback (IPF) can serve as a complementary non-invasive tactile encoding paradigm capable of maintaining effective corrective feedback during repeated human-prosthetic interaction. In contrast to previous studies that primarily emphasized protective perception, withdrawal responses, spatial congruency, or sensory mapping, the proposed approach focuses on the temporal persistence and functional effectiveness of biologically salient tactile stimulation during repeated grasp-control tasks.

Despite notable advances in delivering artificial sensory feedback, common challenges in hand prosthesis use include high cognitive demand, limited intuitive interaction with the environment, reduced perceptual effectiveness of sensory feedback during prolonged human-prosthetic interaction, and extended rehabilitation periods due to the complexity of motor learning and psychosocial adaptation.

Habituation, defined as a gradual decline in neural responsiveness to repeated stimuli, is introduced here as one of the practical motivations for developing alternative sensory feedback modalities rather than as the primary experimental outcome of the present study. From a prosthetic perspective, repeated artificial stimulation may progressively diminish the perceptual salience of sensory cues, particularly in feedback modalities based on repeated vibrotactile or electrotactile stimulation, thereby potentially reducing the effectiveness of sensory feedback during prolonged human-prosthetic interaction. Sensitization, by contrast, refers to an amplified or aversive response to stimulation that may lead to increased discomfort or overstimulation. Sensory adaptation can result from prolonged exposure to consistent and repetitive stimuli, such as electrotactile or vibrotactile inputs. Specifically, habituation can diminish the perceptual salience of such stimuli over time, whereas sensitization may lead to increased discomfort or overstimulation. Both phenomena reflect the nervous system's inherent tendency to attenuate responses to non-novel or non-threatening stimuli, thereby conserving attentional and cognitive resources at the cost of feedback reliability. These limitations contribute to reduced device acceptance, with an estimated 20% of upper-limb amputees discontinuing prosthesis use altogether (Biddiss and Chau, 2007; Simon et al., 2012).

To ensure the effective use of tactile stimulation in prosthetic devices, it is also essential to optimize stimulation parameters to mitigate habituation and preserve perceptual effectiveness during repeated interaction. According to findings in the literature (Graczyk et al., 2016), continuous, unmodulated stimulation is prone to inducing rapid sensory adaptation, diminishing the functional benefit of feedback over time. On the other hand, biomimetic stimulation patterns, which mimic natural sensory encoding mechanisms, can help maintain perceptual stability and reduce the likelihood of sensory fatigue or overstimulation (George et al., 2019). Introducing temporal variability through intermittent, event-driven, or progressively modulated stimulation patterns, as well as adjusting frequency, amplitude, and waveform characteristics, may reduce neural desensitization while preserving perceptual salience during prolonged interaction (D'Anna et al., 2019; Tan et al., 2014). Naturalistic stimulation strategies that emulate the dynamic characteristics of biological tactile signaling have also been associated with improved intuitiveness and reduced cognitive demand, thereby supporting more stable long-term prosthetic interaction (D'Anna et al., 2019). In addition, personalized calibration remains critical because optimal stimulation intensity and preferred stimulation locations vary substantially across individuals. Low-intensity, short-duration stimulation pulses are generally preferred to minimize discomfort and overstimulation (Tan et al., 2014). Closed-loop sensory feedback frameworks capable of adapting stimulation parameters according to task performance and user interaction further highlight the importance of dynamically structured tactile feedback in neuroprosthetic systems (Raspopovic et al., 2014; D'Anna et al., 2019). Collectively, these findings suggest that both the temporal and spatial organization of tactile stimulation play a critical role in maintaining perceptual persistence and functional usability in prosthetic feedback systems.

Motivated by these limitations, the current study investigates the impact of innocuous painful stimulation on virtual prosthetic grasp control, with particular emphasis on the persistence of feedback effectiveness during repeated interaction. Habituation and sensitization provide the broader sensory-adaptation context motivating the stimulation strategies investigated in this study; however, these perceptual phenomena are not directly quantified as experimental outcomes. By exploring a biologically salient mechanonociceptive feedback modality, the research aims to improve grasp-control performance and the functional effectiveness of sensory feedback in upper-limb prosthetic systems. Understanding how such feedback influences control accuracy and long-term interaction stability requires consideration of sensory processing and adaptation mechanisms within the human somatosensory system.

Within this framework, the study proposes a biologically salient tactile feedback strategy based on intermittent, low-amplitude mechanical stimulation delivered through a spherically tipped cone tactor to provide perceptually distinct tactile cues while minimizing discomfort and perceptual fatigue. Unlike conventional electrotactile or vibrotactile methods, the proposed approach utilizes mechanonociceptive tactile stimulation that remains perceptually effective yet tolerable during repeated interaction, with the goal of maintaining perceptual salience while minimizing the risk of overstimulation. In addition, the feedback is dynamically modulated in response to task-related errors in real time, ensuring variability in stimulation patterns, which may help reduce sensory adaptation and preserve interaction stability during prolonged use. By targeting a stimulation modality that is both perceptually stable and physically valid, the study aims to offer a practical solution for improving prosthetic function without the common trade-offs associated with current sensory substitution systems.

## 2 Materials and methods

The study is structured into three distinct phases: experimental setup preparation, initial evaluation, and feedback optimization. In the first phase, a specialized experimental setup is developed and configured to simulate the sensory and motor conditions experienced by individuals with upper extremity amputations during object interaction tasks, such as grasping a cylindrical item. This setup enables the use of healthy participants while ensuring that the experimental conditions closely mimic those encountered in prosthetic control, particularly in the absence of natural somatosensory feedback. Within this framework, participant actions and feedback-driven corrections form a human-in-the-loop interaction process, enabling controlled investigation of feedback-based motor adaptation. In the second phase, the performance of innocuous painful stimulation is systematically assessed in comparison to conventional feedback modalities, namely visual and force feedback, by analyzing task accuracy and control performance. Specifically, force feedback is selected as the tactile comparator due to its conceptual proximity to innocuous painful stimulation in conveying error-related information through mechanical force application. Building upon these results, the third phase focuses on a detailed characterization of IPF, aiming to optimize stimulation parameters such as frequency, temporal modulation, and spatial configuration to enhance both user performance and perceptual salience.

To accurately simulate prosthetic usage conditions in a controlled setting, experiments were conducted with a custom-designed experimental platform involving eight healthy adult volunteers (*n* = 8; four males, four females; age 25.4 ± 7.4 years; all right-handed). The study was designed as an exploratory proof-of-concept investigation to characterize the feasibility and behavioral effects of the proposed IPF approach under controlled human-in-the-loop conditions. A within-subject experimental design was employed to enable direct comparisons across experimental conditions while reducing the influence of inter-individual variability. Participants were recruited over the period from 07/01/2025 to 07/15/2025. This study was approved by the Ethics Committee of Istanbul Medipol University (approval number: E-10840098-202.3.02-4105). All participants provided written informed consent prior to participation. All eight participants completed the experimental protocol, with no stimulation-related withdrawals or premature termination of experimental sessions. No adverse events associated with IPF stimulation were observed or reported during either stimulus adjustment or the main experiments. This setup replicates key aspects of the sensory-motor experience of individuals with upper limb loss, particularly during object interaction tasks such as grasping a cylindrical item, thereby enabling relevant and translatable findings.

### 2.1 Experimental setup

The experimental setup comprises three distinct sections: a hand exoskeleton, a **tac**tile stimula**tor** (a.k.a. tactor), and a virtual reality (VR) environment (see Figure 1). The key role of the hand exoskeleton is to accurately convey the natural movements of the middle finger and thumb to the VR environment while attempting to grasp an object. The VR environment visually represents the position of the finger, providing clear guidance to the volunteer, together with a virtual object whose diameter varies randomly. Meanwhile, the tactor delivers targeted tactile stimulation to the volunteer's upper arm to correct misalignment between the finger position and the boundary of the virtual object.

**Figure 1:**
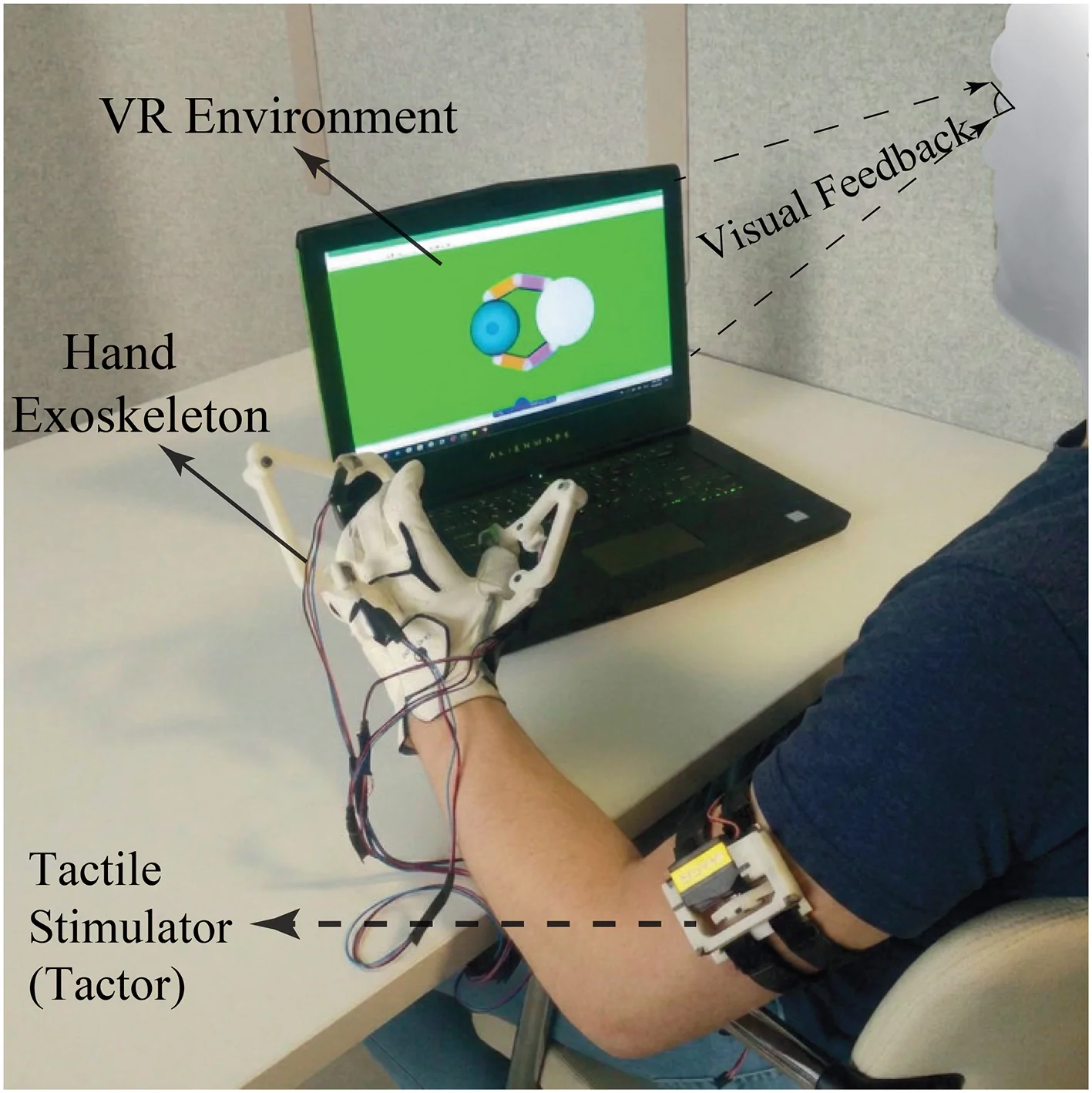
Overall experimental setup used throughout the study. The participant performed the virtual grasping task using the linkage-based hand exoskeleton while interacting with the virtual reality environment through visual feedback. Tactile feedback was delivered by the upper-arm stimulator (tactor), providing the sensory feedback evaluated in the subsequent experiments.

#### 2.1.1 Hand exoskeleton design

The key role of the hand exoskeleton device is to determine the fingertip position of the middle finger and thumb during pinch grasping. In particular, the two-finger linkage-based exoskeleton mounted on the glove is equipped with high-precision potentiometers. Two miniature single-turn linear rotary potentiometers are mechanically coupled to the thumb and middle-finger linkage mechanisms to provide continuous analog measurements of the joint angles. These potentiometers provide continuous analog measurements of the exoskeleton joint angles, which are subsequently used for real-time fingertip position estimation. This type of design is also present in the literature (Battezzato, 2014; Iqbal et al., 2014; Asran et al., 2025). In this study, the hand exoskeleton system, a subsystem of the experimental setup, is highlighted not for its design aspect but for its role in accurately measuring the positional changes of the fingers with high precision. While potentiometers mounted directly on the finger joints of a glove cannot accurately detect characteristic information such as angular changes at the finger joints and the initiation of motion, utilizing an exoskeleton expands the movement space, enabling the detection of even the smallest-scale movements with high accuracy. The high-precision rotary potentiometers measure the angular displacements of the thumb and middle-finger exoskeleton joints, as shown in Figure 2. The analog signals are digitized in real time using the 10-bit analog-to-digital converter of the Arduino^®^ Mega 2560 microcontroller, providing 1024 discrete quantization levels over the measurement range. The digitized signals are subsequently converted into joint-angle measurements. The fingertip position is then determined from these measured joint angles using an analytical vector-loop-based forward kinematic model of the closed-linkage exoskeleton. The computed fingertip position is subsequently transmitted to the VR environment for real-time control of the virtual gripper. The forward kinematic formulation establishes the geometric relationship between the measured exoskeleton joint angles and the corresponding fingertip position without employing small-angle or iterative numerical approximations. Consequently, the reported position error is obtained from the analytically computed fingertip position derived from the measured exoskeleton joint angles. Because the finger position is computed analytically from the joint-angle measurements, no additional approximation error is introduced by the kinematic computation itself. Furthermore, no measurable mechanical backlash was observed within the operating range of the exoskeleton during the experiments. Therefore, the principal sources affecting the estimated position error are the accuracy of the joint-angle measurements and the geometric parameters of the exoskeleton rather than approximation errors associated with the forward kinematic model. This configuration allows participants to control a virtual gripper through a hand exoskeleton interface. To preserve the natural biomechanics of finger movement during task execution, the linkage components of the exoskeleton are fabricated from lightweight, high-impact polystyrene, providing structural durability while minimizing motion interference.

**Figure 2:**
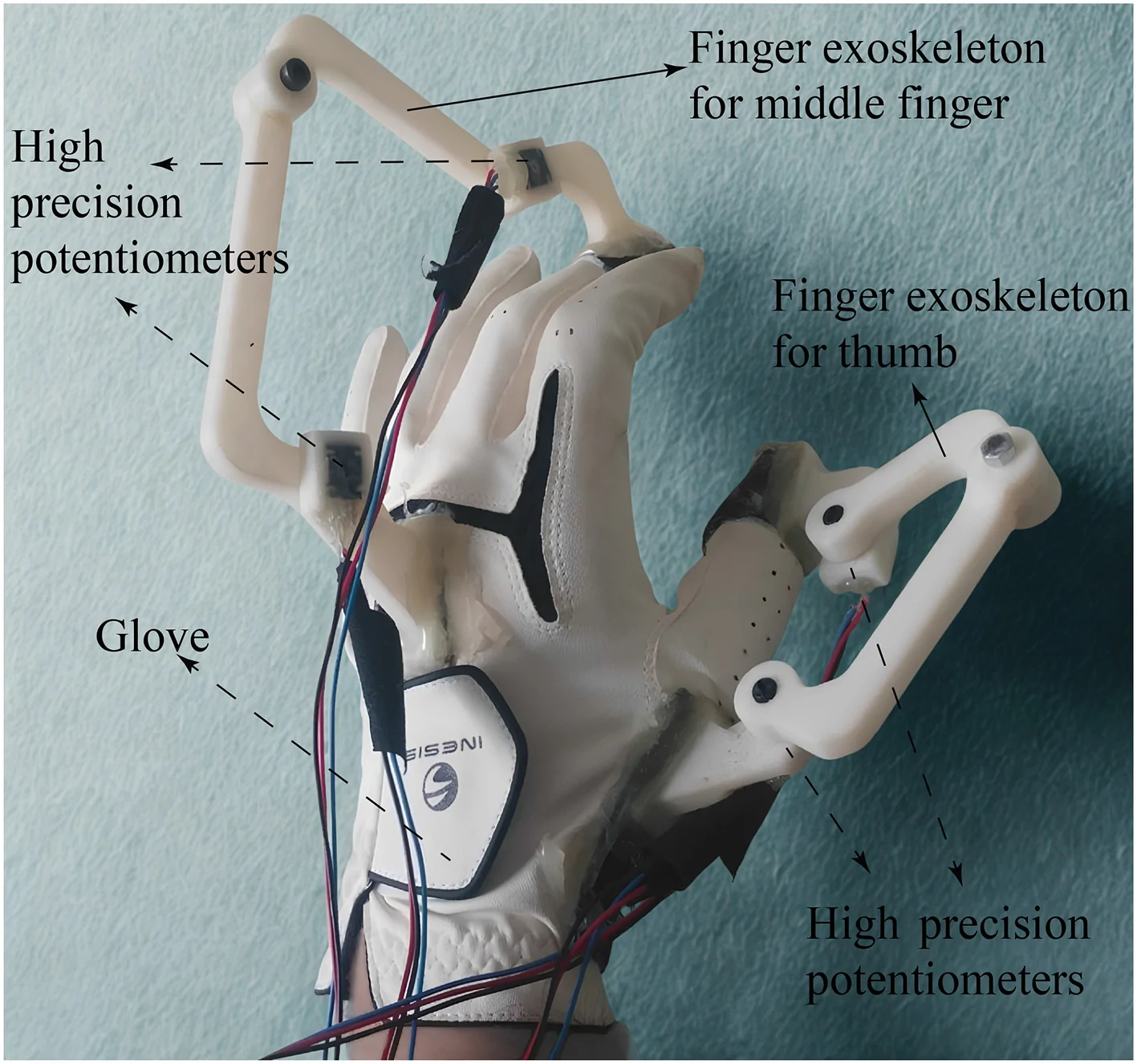
Linkage-based two-finger hand exoskeleton used to control the virtual grasping task. The device is secured to a glove and incorporates independent thumb and middle-finger mechanisms equipped with high-precision potentiometers for finger-position measurement.

#### 2.1.2 Virtual reality (VR) environment design

The VR environment is designed to mimic the realistic situation of amputees who lack somatosensory feedback when holding an object with a hand prosthesis and therefore rely predominantly on visual feedback. As represented in Figure 3, the VR environment primarily consists of two key components: a virtual gripper that simulates the motion of the middle finger and thumb, and a virtual cylindrical object representing a bottle. During the experiments, the radius of the target object model dynamically and randomly changes over time, requiring the volunteer to grasp it using the hand exoskeleton device. The success criterion of grasping the virtual object model in the VR environment is assessed based on the amount of intrusion of the finger into the boundary of the cylindrical object, as illustrated in Figure 4. The level of intrusion is continuously calculated and monitored as an error magnitude throughout the task.

**Figure 3:**
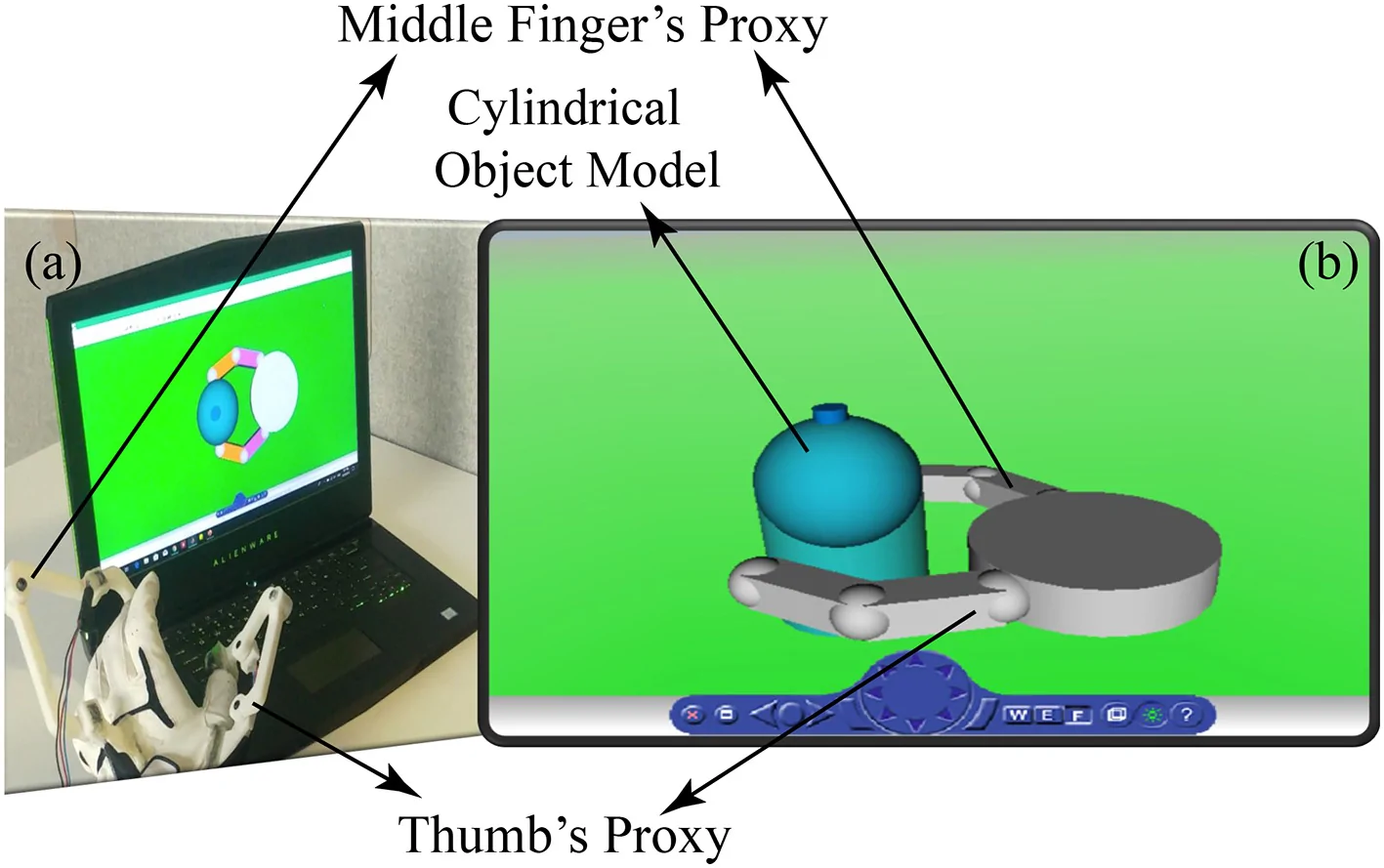
VR environment used throughout the experiments. **(a)** Experimental setup during the grasping task. **(b)** Isometric view of the virtual scene, showing the thumb and middle-finger proxies and the cylindrical virtual object.

**Figure 4:**
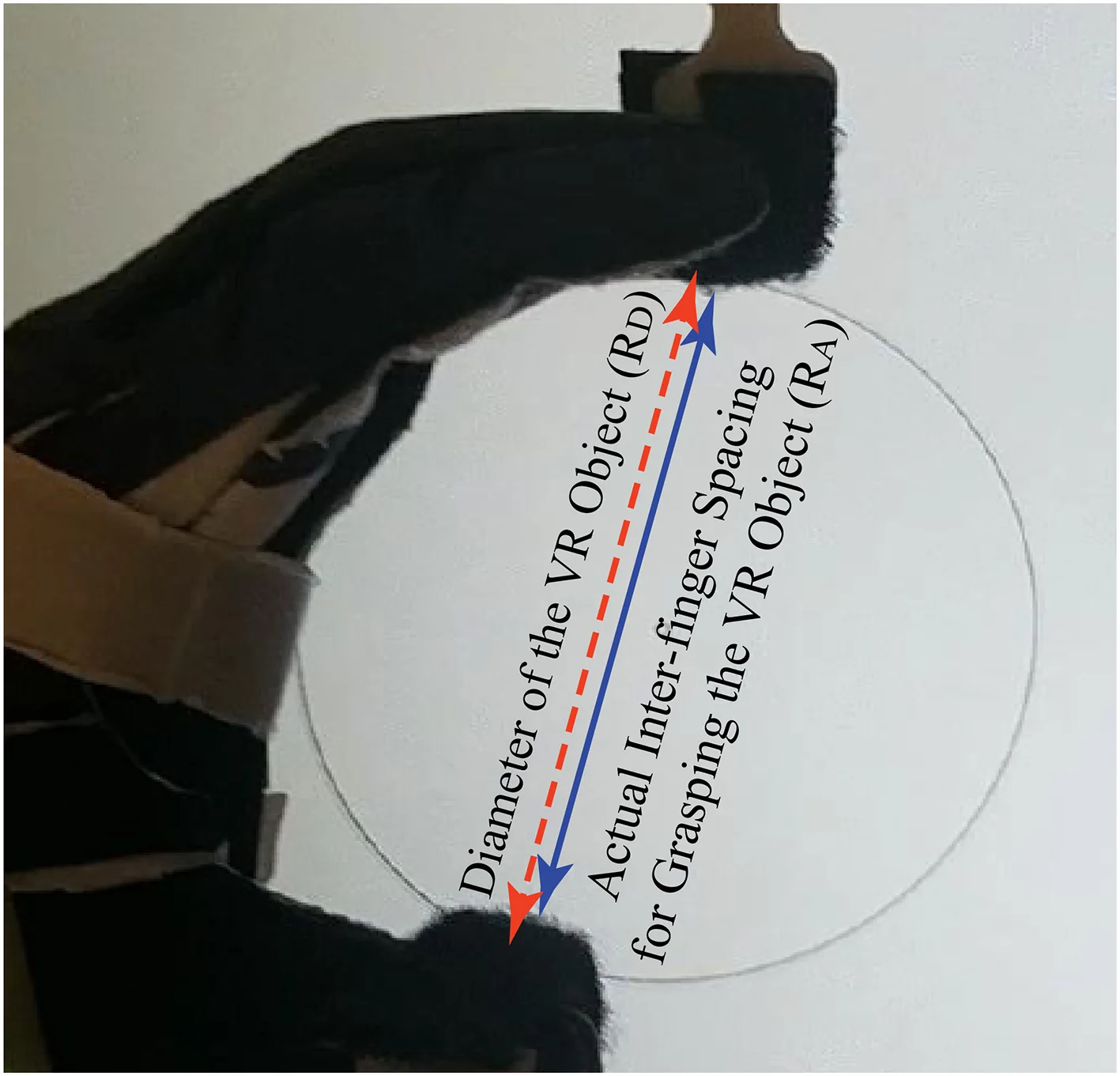
Demonstration of fingertip penetration beyond the boundary of the virtual object during grasping. Penetration occurs when the actual inter-finger spacing (*R*_*A*_) exceeds the virtual object diameter (*R*_*D*_). The resulting penetration depth is defined as the position error used throughout the experiments to quantify grasp-control performance.

The *position error* *E*(*t*) represents the amount of intrusion of the virtual middle fingertip into the boundary of the virtual cylindrical object at a given time *t*. The error is computed as shown in Equation 1:


$$\begin{array}{l} E\left(t\right)=\max\left(0,r_{d}\left(t\right)-r_{o}\left(t\right)\right) \end{array}$$ (1)

where:

- *r*_*o*_(*t*) is the radial position of the middle fingertip from the central axis of the virtual cylindrical object at time *t*,
- *r*_*d*_(*t*) is the radius of the virtual object at time *t*,
- *E*(*t*) = 0 indicates no intrusion (i.e., the finger is on or outside the surface),
- *E*(*t*) > 0 indicates intrusion into the object, representing a control error.

Both *r*_*o*_(*t*) and *r*_*d*_(*t*) are defined in the radial coordinate frame centered at the cylindrical object's axis, allowing the penetration depth to be computed geometrically from the difference between the fingertip radial distance and the object radius.

The zero in the expression ensures that only intrusions into the object are treated as errors, preventing the calculation of negative, non-physical values when the finger does not penetrate the virtual object. The stimulation is triggered based on the error computed over discrete, non-overlapping 100 ms time windows. For a task consisting of *N* such windows, the mean position error is defined as shown in Equation 2:


$$\begin{array}{l} \overset{-}{E}=\frac{1}{N}\overset{N}{\underset{i=1}{\sum }}E_{i} \end{array}$$ (2)

where *E*_*i*_ is the maximum position error observed within the ( *i*th) 100 ms window.

This temporal windowing strategy allows the stimulation controller to respond to sustained intrusion events rather than instantaneous measurement noise. This window-based calculation dynamically determines the stimulation duration in real time, based on the persistence of the error during the task.

#### 2.1.3 Tactile stimulator (tactor) design

The tactile stimulator was designed to provide wearable mechanical feedback for individuals with upper-extremity amputations. Within the framework of this study, innocuous painful feedback (IPF) was compared with a conventional non-painful force-feedback condition to convey information regarding the instantaneous grasping state.

The mechanical tactor tip was actuated using a SAVÖX SH-1357 digital mini servo incorporating an internal closed-loop position controller. Accordingly, the stimulator was implemented as a position-controlled mechanical stimulation system rather than a force/torque-controlled system. Stimulation intensity was adjusted by modifying the commanded servo reference position. The tactor tip was rigidly coupled to the servo output without intermediate transmission elements such as tendons, cables, compliant mechanisms, or flexible linkages, thereby minimizing additional transmission-induced compliance, backlash, and dynamic delay. The complete system was secured to the participant's upper arm using adjustable Velcro^®^ straps, as shown in Figure 5a.

**Figure 5:**
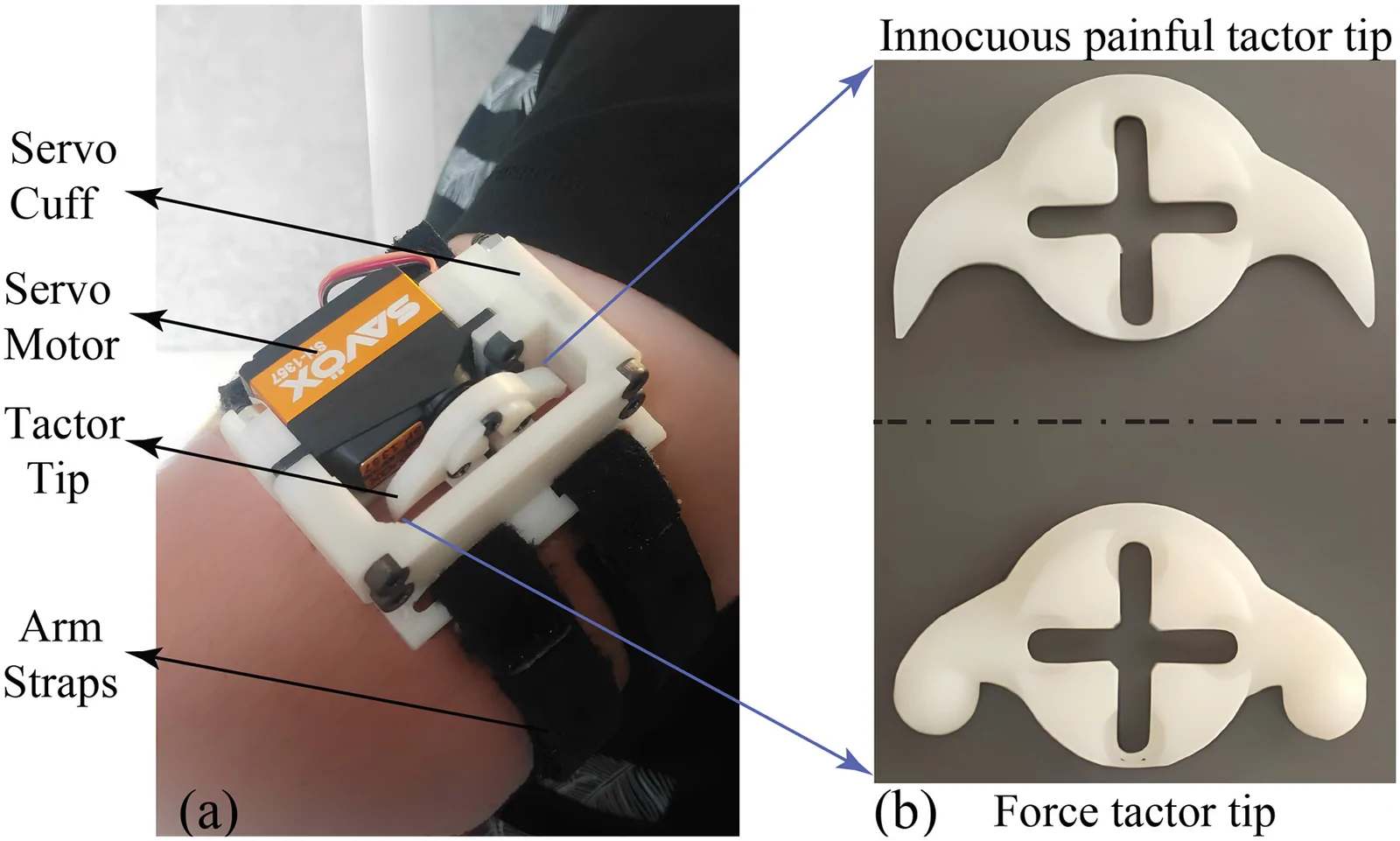
Tactile stimulator (tactor): **(a)** fabricated device mounted on the participant's upper arm and **(b)** replaceable tactor tips used for innocuous painful stimulation and non-painful force feedback.

Two interchangeable tactor-tip geometries were used: a conical tip with a point diameter of 1 mm for IPF and a spherical tip with a diameter of 6 mm for non-painful force feedback. The geometries were selected during device development to generate clearly distinguishable localized mechanonociceptive and diffuse pressure sensations while maintaining suitability for repeated stimulation. Reducing the conical-tip diameter concentrates the applied load over a smaller contact region and increases local contact stress. However, accurate analytical prediction of the interaction between a rigid conical tip and compliant human skin is difficult because of the nonlinear and subject-dependent mechanical behavior of soft tissue. The conical-tip diameter was therefore progressively reduced during device development until the smallest geometry that consistently produced a localized mechanonociceptive sensation without excessive skin indentation or noticeable transient skin marks during repeated stimulation was identified. This selection is consistent with evidence that smaller and sharper contact geometries produce more localized sharp sensations and more readily evoke mechanically induced cutaneous pain, whereas larger contact geometries require greater stimulation to elicit comparable percepts (Greenspan and McGillis, 1991). Punctate mechanical stimulation is also an established method for assessing mechanical pain sensitivity in standardized quantitative sensory testing protocols (Rolke et al., 2006). The 1-mm conical tip was therefore retained for IPF, whereas the 6-mm spherical tip was selected to distribute the applied load over a broader contact region and produce a diffuse, non-painful pressure sensation for the force-feedback condition. Both interchangeable tactor tips were intentionally designed with identical overall heights. Consequently, replacing the conical tip with the spherical tip did not alter the participant-specific contact geometry or the commanded servo reference position established during the initial contact calibration. Therefore, both feedback modalities shared the same calibrated actuator position, and the comparison isolated the effect of contact geometry rather than differences in actuator positioning or contact configuration.

As illustrated in Figure 5b, each replaceable tip contains applicator surfaces on both sides of the rotating component. Clockwise rotation provides stimulation at one skin site, whereas alternating clockwise and counterclockwise rotations enable sequential stimulation at two spatially distinct sites (dual skin sites).

The sharp-tipped stimulator was additionally characterized on a benchtop using a load cell. Under the characterization condition, the measured contact force was converted to an estimated nominal contact pressure of 0.94 ± 0.13 N/mm^2^. This value was calculated by dividing the measured contact force by the nominal circular area of the 1-mm-diameter tip. The reported nominal contact pressure provides a mechanical characterization of the stimulation delivered at the calibrated operating servo position. The term *nominal* is used because the actual tip–skin contact area cannot be assumed to remain equal to the geometric tip area owing to local skin deformation. This load-cell measurement characterizes the mechanical output of the device and should not be interpreted as a pressure that was directly controlled or applied identically to every participant. During the human experiments, pressure was neither measured in real time nor regulated through closed-loop force feedback.

Prior to threshold identification, a one-time contact calibration was performed for each participant. No force or tactile sensor was incorporated into the wearable tactor for real-time detection of skin contact. Instead, the position-controlled servo was gradually advanced until stable initial contact between the tip and the skin was visually confirmed. The corresponding commanded servo position was then defined as the participant-specific reference contact position. At this position, the participant typically perceived initial mechanical contact, but this contact position was not regarded as the pain threshold.

Starting from the calibrated contact position, the lower and upper perceptual bounds were established using a calibration procedure based on the psychophysical principle of the ascending method of limits (Gescheider, 1997). The commanded servo position was increased in approximately 1° increments over a limited angular range until the participant first reported the onset of pain. The corresponding servo reference position was recorded as the lower bound, Level *L*. The position was then increased further using the same incremental procedure until the participant first reported the onset of severe pain, and this reference position was recorded as the upper bound, Level *H*. The limited angular range and small increments enabled gradual adjustment of skin indentation while avoiding abrupt increases in stimulation. The resulting *L* and *H* values were calibration variables expressed in terms of commanded servo position; they were not treated as direct pressure measurements or experimental outcome variables. Accordingly, the objective of this procedure was to establish participant-specific operating stimulation levels rather than to perform a formal psychophysical threshold study.

Previous work has demonstrated the value of subject-specific calibration in sensory interfaces by adapting stimulation parameters to individual perceptual thresholds (Isaković et al., 2019). Motivated by this principle, the operating reference position used during the main experiment was calculated from the individually determined lower and upper bounds according to Equation 3:


$$\begin{array}{l} I=L+\alpha \left(H-L\right) \end{array}$$ (3)

where *I* denotes the commanded servo reference position used for stimulation. The scaling factor α was set to 0.5, corresponding to the midpoint between the participant-specific pain-onset position *L* and severe-pain-onset position *H*. This intermediate operating point was selected as a practical compromise between perceptual salience and participant comfort, providing a clearly perceivable mechanonociceptive stimulus while avoiding excessive nociceptive activation and reducing the likelihood of accumulating discomfort during repeated stimulation. Participants were instructed to immediately report excessive pain or discomfort during both the calibration procedure and the main experimental sessions, and stimulation could be terminated at any time at the participant's request. During calibration, stimulation intensity was not increased beyond a level considered tolerable by the participant. In the main experiments, the participant-specific operating position *I*, established during calibration, was maintained without further increases in stimulation intensity.

Following calibration, the resulting servo reference position *I* was stored and reused throughout all experimental trials for the same participant without adaptive modification. The same commanded servo reference position was reproduced by the servo's internal closed-loop position controller, thereby providing consistent participant-specific stimulation conditions across repeated trials. Because the system operates under position control rather than force control, contact pressure is not directly controlled, and a fixed relationship between a 1° position increment and contact pressure cannot be assumed. Consequently, the mechanical stimulation produced at a given commanded servo reference position depends on the interaction between the rigid tactor, the compliant skin, the mounting condition, and the actuator dynamics, rather than on the commanded position alone.

During stimulation, the tactor operated about the calibrated contact position using small-amplitude sinusoidal angular oscillations. Therefore, the actuator was not required to repeatedly execute large-angle positioning movements or re-establish contact from a distant initial position. The tested stimulation frequencies of 5, 30, and 60 Hz corresponded to the frequency of these localized mechanical oscillations about the calibrated operating position. The combination of small angular excursions and rigid coupling between the servo output and tactor tip limited the required actuator displacement and minimized additional transmission-related dynamic effects at the higher commanded stimulation frequencies.

The mechanical components of the tactile stimulator were fabricated from ABS because of its low mass and mechanical durability. The complete tactor unit, including the SAVÖX SH-1357 digital mini servo, weighed 26 g, enabling prolonged upper-arm placement without substantial mechanical burden. Simulink^®^ 3D Animation™ (The MathWorks, Inc., Natick, MA, United States), MATLAB^®^ (The MathWorks, Inc., Natick, MA, United States), and the Arduino IDE were used to initiate stimulation, specify the commanded amplitude and frequency, record the middle-finger and thumb positions, and transmit these measurements to the virtual-reality environment.

### 2.2 Experimental procedure

In the context of these experiments, several critical questions are addressed, each aimed at unraveling the efficacy and characteristics of the innocuous painful stimulation. The experimental sequence is deliberately arranged to begin with the identification of the stimulation type, followed by the characterization phase aimed at determining the optimal feedback stimulation.

*Question-I: Does the IPF elicit a reflex-like response, which, when combined with voluntary movement, enhances position control more effectively than visual feedback alone or in conjunction with force feedback?*

Before data collection, participants received standardized task instructions describing the objective of the experiment and the required grasping task. During device preparation, the interchangeable force-feedback and innocuous painful tactor tips were demonstrated so that participants understood the two feedback modalities before testing. Because the experimental task consisted only of grasping a virtual circular object while maintaining the gripper within its boundary, no separate practice trials were performed prior to data collection. To examine this problem, the experiment is structured into three distinct sessions. In the first session, the volunteer relies exclusively on visual feedback to position their fingers and grasp the virtual object (see Figure 1). Throughout all experiments, the virtual object remained circular, whereas its diameter was randomly varied from trial to trial to prevent anticipatory grasping strategies and minimize potential learning effects. In the second session, the tactor is worn on the upper arm, delivering force feedback to the subject when the gripper exceeds the object's boundary. The third and final session focuses on evaluating the effects of IPF on the same task. Consequently, the experiment consists of three main sessions: the first assesses visual feedback alone, the second combines visual and force feedback, and the third integrates visual feedback with IPF. Each session comprises three consecutive subsessions, with each subsession consisting of seven trials as depicted in Figure 6. The duration of each session is 240 s, including 30-s rest periods. Additionally, there is a 60-s break between sessions. These rest periods are incorporated to minimize cumulative stimulation, reduce participant discomfort associated with repeated mechanonociceptive stimulation, and help limit potential short-term habituation associated with repeated cutaneous stimulation, thereby maintaining the perceptibility of the feedback throughout the experimental sessions (Milne et al., 1991).

**Figure 6:**
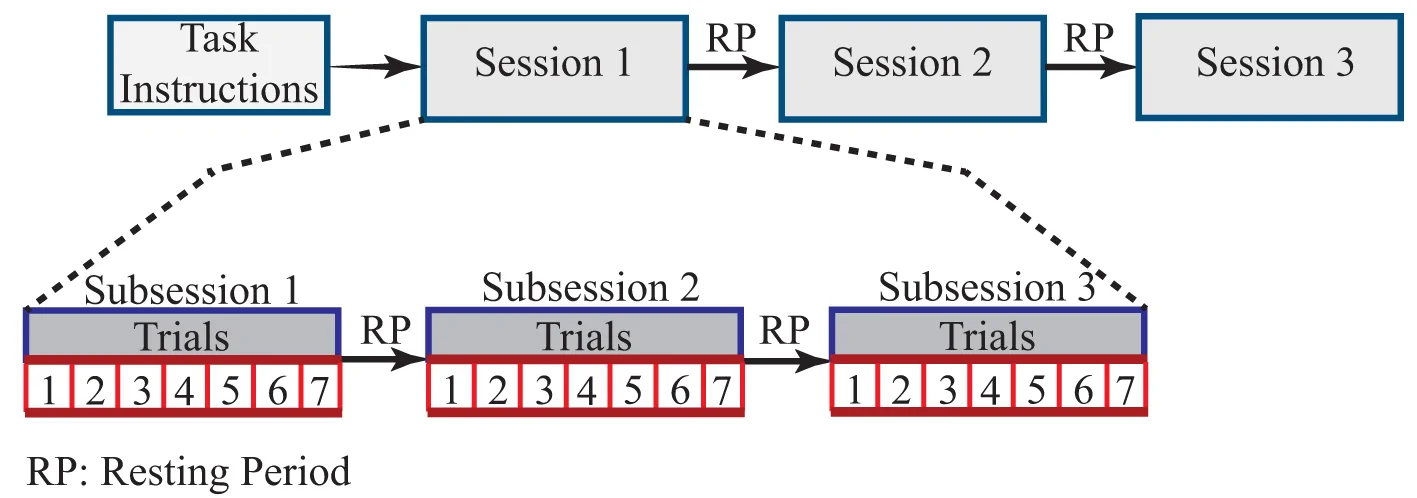
Experimental procedure for Experiment 1, comparing the effectiveness of three feedback modalities (Visual-only, Visual + Force, and Visual + IPF) during virtual prosthetic grasping. Participants completed repeated grasping trials under each feedback condition, and grasp-control performance was quantified using the position error.

*Question-II: Does the frequency of IPF impact the precision of position control in a prosthetic hand?*

The second experiment was designed to investigate the effect of stimulation frequency on grasp-control performance. Three predetermined stimulation frequencies representing low (5 Hz), intermediate (30 Hz), and high (60 Hz) temporal stimulation conditions were evaluated. These predetermined frequency levels were fixed for all participants and represented low-, intermediate-, and high-frequency stimulation conditions within the practical operating range of the proposed stimulator, whereas only the stimulation intensity was individualized through the participant-specific calibration procedure described in Section 2.1.3.

The selection of the investigated frequency range was additionally motivated by the frequency-dependent characteristics of cutaneous tactile processing. Different temporal stimulation regimes are associated with distinct perceptual characteristics and patterns of rapidly adapting tactile afferent activity (Ng et al., 2021), and stimulation frequency has been identified as an important psychophysical design parameter in non-invasive vibrotactile feedback for upper-limb prosthetic interfaces (Karakuş and Güçlü, 2020). Accordingly, 5 Hz was selected to represent a clearly separated low-frequency stimulation condition, 30 Hz was included as an intermediate comparison frequency, and 60 Hz represented the high-frequency condition within the investigated range. Thus, the selected frequencies were intended to sample distinct temporal stimulation conditions within both a physiologically relevant tactile range and the practical operating range of the proposed stimulator, rather than to selectively target individual mechanoreceptor classes.

This design enabled us to evaluate whether grasp-control performance changed systematically with increasing stimulation frequency while limiting the number of experimental conditions and isolating frequency as the primary independent variable. Among the evaluated frequencies, 60 Hz was selected as the highest frequency that could be reliably generated by the proposed stimulator while maintaining localized mechanical stimulation about the calibrated contact position during repeated stimulation. Before each experiment, the stimulator was calibrated individually to determine the participant-specific operating stimulation level. Each stimulation frequency was evaluated in a single 80-s subsession consisting of a 20-s preparation period followed by a 60-s experimental period, as illustrated in Figure 7. The order of the three frequency conditions was randomized across participants to minimize potential order and habituation effects (Paredes et al., 2015).

**Figure 7:**
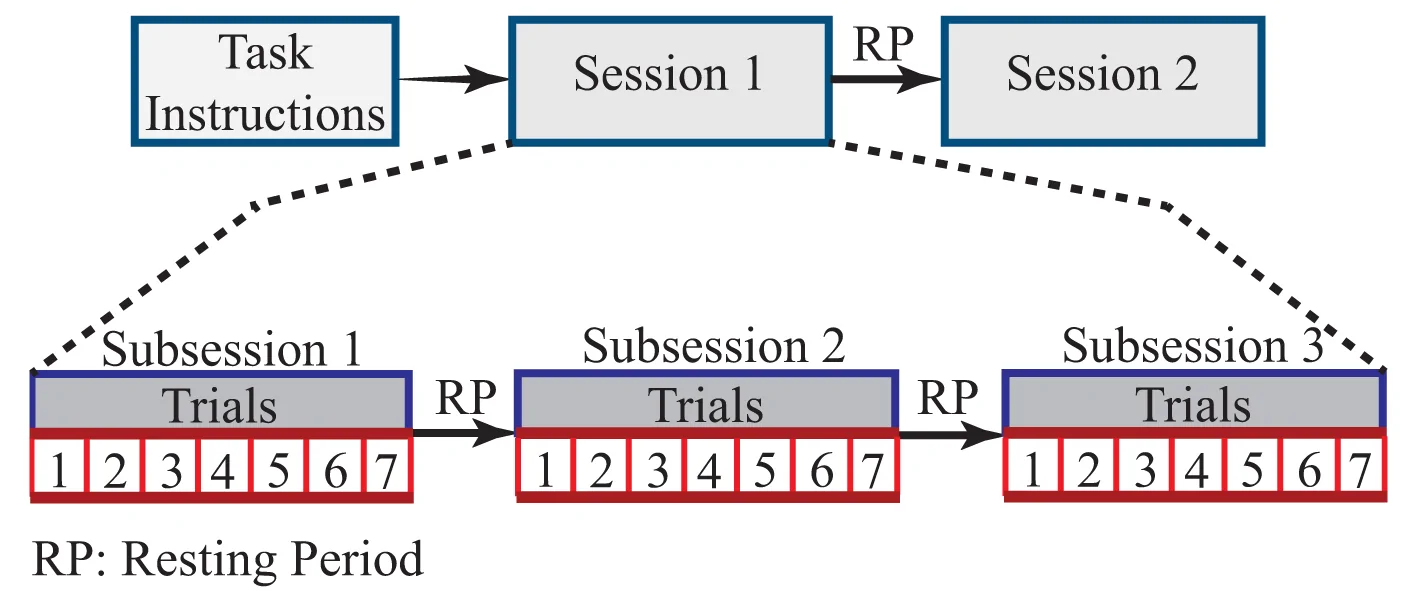
Experimental protocol adopted for Experiment 2. Three predetermined IPF stimulation frequencies (5, 30, and 60 Hz) were evaluated to investigate the effect of stimulation frequency on grasp-control performance.

*Question-III: What if we gradually increase the frequency based on the error rate?*

Habituation and sensitization represent interdependent changes in the perceptual response to repeated stimulation (Barry, 2012). Repeated stimulation may lead to short-term habituation and thereby reduce the perceptual salience of tactile feedback over time (Thompson, 2009), whereas sensitization may amplify the response to sufficiently intense stimuli. Accordingly, an effective tactile-feedback strategy should preserve stimulus perceptibility during repeated interaction without continuously exposing the user to the highest stimulation frequency. The preceding experiments characterized the efficacy of IPF and the influence of stimulation frequency. Experiment III therefore investigated whether adjusting the stimulation frequency according to the instantaneous position error could maintain grasp-control performance more effectively than constant high-frequency stimulation during repeated interaction.

The fixed 60-Hz condition identified in Experiment II was used as the reference condition. In the Progressive Frequency Modulation (PFM) condition, the stimulation frequency was not varied continuously; instead, it was selected from the three predetermined frequency levels evaluated in Experiment II: 5, 30, and 60 Hz. Let *e* denote the instantaneous absolute position error in millimeters. The error-dependent frequency-selection rule was defined as shown in Equation 4:


$$\begin{array}{l} f\left(e\right)=\{\begin{array}{ll} 5\text{ Hz}, & e<0.8\text{ mm},\\ 30\text{ Hz}, & 0.8\text{ mm}\leq e<1.2\text{ mm},\\ 60\text{ Hz}, & e\geq 1.2\text{ mm}. \end{array} \end{array}$$ (4)

Thus, small, intermediate, and large position errors activated the low-, intermediate-, and high-frequency stimulation levels, respectively. PFM was therefore implemented as a deterministic, error-dependent discrete frequency-switching strategy. The term *progressive* refers to the increase in stimulation frequency with increasing error magnitude, rather than to a gradual increase in frequency over elapsed time. The comparison was evaluated under two repeated-interaction protocols comprising 15 consecutive trials for the short-term evaluation and 35 consecutive trials for the extended evaluation. This experiment compared the error-dependent PFM strategy with constant 60-Hz stimulation to determine whether adapting the temporal characteristics of the feedback to task error could better preserve feedback effectiveness during both short-term and extended repeated interaction.

*Question IV: Does the spatial stimulation configuration (single-site vs. sequential dual-site stimulation) influence the efficacy of position control?*

Since the effectiveness of tactile feedback depends not only on its temporal characteristics but also on how the stimulation is spatially delivered, the spatial stimulation configuration represents an important design variable in human–machine interaction. In upper-limb prosthetic systems, efficient position control depends on delivering sensory information in a manner that enables the user to rapidly detect and correct grasping errors. Accordingly, this experiment investigates whether spatial stimulation configuration influences position-control performance during prosthetic grasping by comparing single-site and sequential dual-site stimulation, as shown in Figure 8.

**Figure 8:**
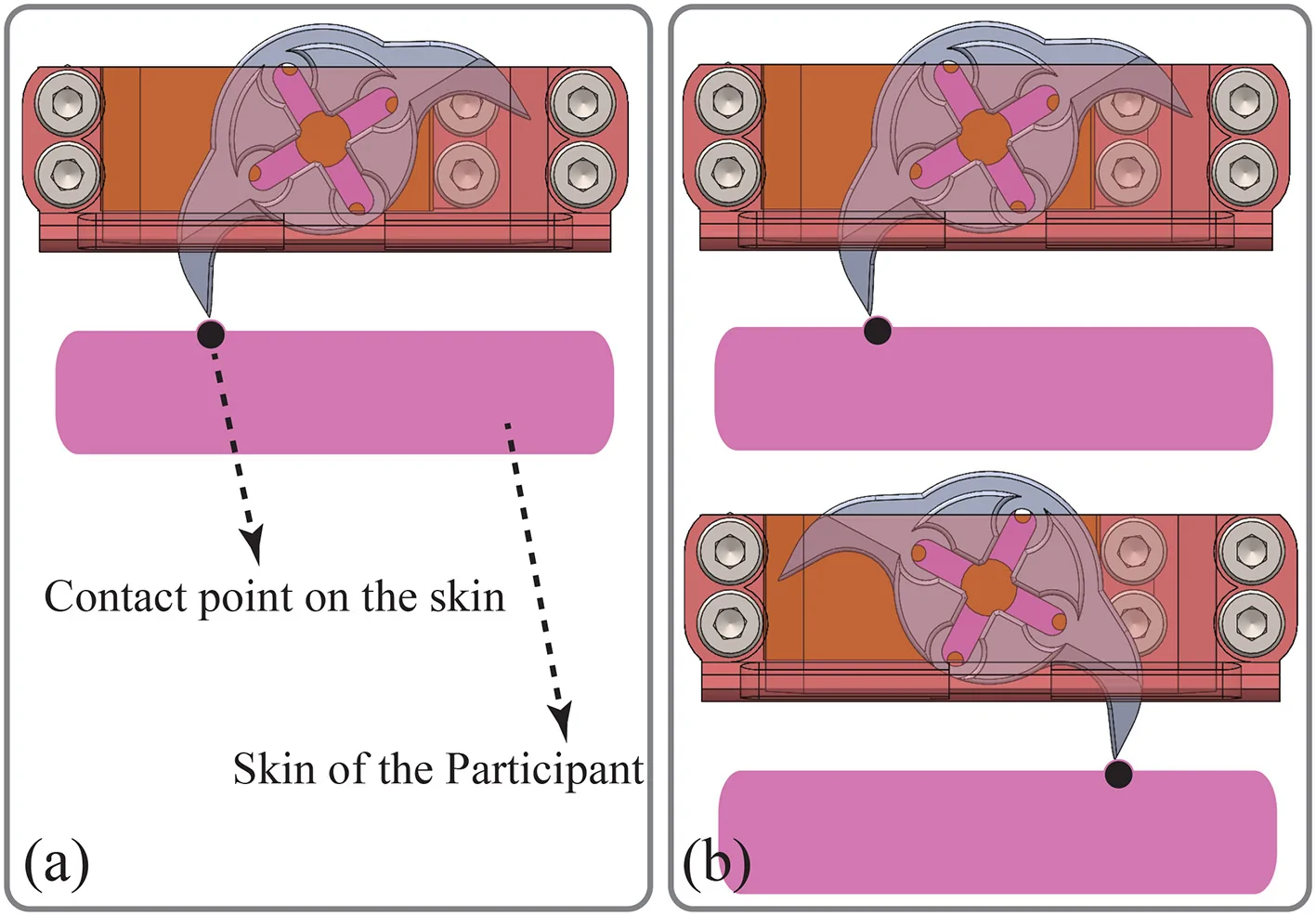
Schematic illustration of the two spatial stimulation configurations evaluated in Experiment 4: **(a)** single-site stimulation, in which repeated stimulation is delivered to a single calibrated skin-contact location, and **(b)** sequential dual-site stimulation, in which the stimulator alternates between two calibrated skin-contact locations while maintaining identical stimulation frequency and actuator motion.

The two stimulation configurations were applied to the upper arm under otherwise matched experimental conditions. The servo actuator generated small-amplitude sinusoidal angular oscillations about the calibrated contact position to maintain consistent skin contact throughout stimulation. Because the interchangeable tactor tips were designed with identical overall heights, replacing one tip with the other did not alter the calibrated servo reference position established during the contact calibration procedure. Both stimulation sessions used 60-Hz feedback but differed only in spatial configuration: Session 1 applied stimulation at a single site, whereas Session 2 involved stimulation at two separate sites in sequence. Thus, stimulation frequency, actuator motion, and the experimental protocol were maintained across the two conditions, while the spatial stimulation configuration was varied. Each stimulation configuration was evaluated over 20 consecutive trials.

## 3 Experimental evaluations

The experimental evaluations presented in this section are designed to systematically address the four characterization questions introduced earlier. For clarity and coherence, each experimental evaluation is presented sequentially under its respective subheading. All statistical analyses were performed using MATLAB (2025, MathWorks, USA). Statistical analyses were performed on participant-level data (*n* = 8) or using repeated-measures models that explicitly accounted for observations nested within participants. Statistical methods were selected according to the design and hypotheses of each experiment: Experiment 1 used paired Student's (*t*)-tests for predefined within-subject comparisons, Experiment 2 used the Friedman test followed by Wilcoxon signed-rank tests for pairwise comparisons, and Experiments 3 and 4 used linear mixed-effects models (LMMs) to account for repeated observations within participants. Where multiple predefined pairwise comparisons were performed within the same family of hypotheses, Holm's sequential correction was applied to control the family-wise error rate. Statistical significance was defined as (*p* < 0.05), and effect sizes are reported where appropriate.

### 3.1 Experiment 1: Effectiveness of IPF in enhancing position control compared to visual and force feedback

The present study investigated the influence of feedback modalities on grasping performance, with particular attention to trial-by-trial performance dynamics during repeated interaction. The efficacy of three feedback conditions, *visual-only, visual + force*, and *visual + IPF*, was evaluated in terms of motor performance across repeated practice. The order of the three feedback conditions (Visual-only, Visual + Force, and Visual + IPF) was randomized across participants to minimize potential order, practice, and fatigue effects. Within each condition, however, the seven trials of a subsession were completed consecutively under the same feedback modality before the participant proceeded to the next condition.

Two complementary analyses were conducted: (i) subsession-wise distributions with median trends and paired comparisons, highlighting trial-to-trial variability and within-condition performance trends, and (ii) participant-wise comparisons across feedback conditions, summarizing overall performance differences between modalities.

The results showed that feedback modality strongly shaped task performance (see Figure 9). In particular, *visual-only feedback* produced the highest error values, indicating that visual information alone was insufficient for accurate grasp control. The addition of *visual + force feedback* reduced the error, underscoring the importance of multimodal reinforcement in sensorimotor regulation. The greatest improvement occurred with *visual + innocuous painful feedback (IPF)*, where the mean error stabilized around 3 mm. These results suggest that IPF serves not only as a corrective signal but also as a highly salient feedback channel that promotes rapid error reduction.

**Figure 9:**
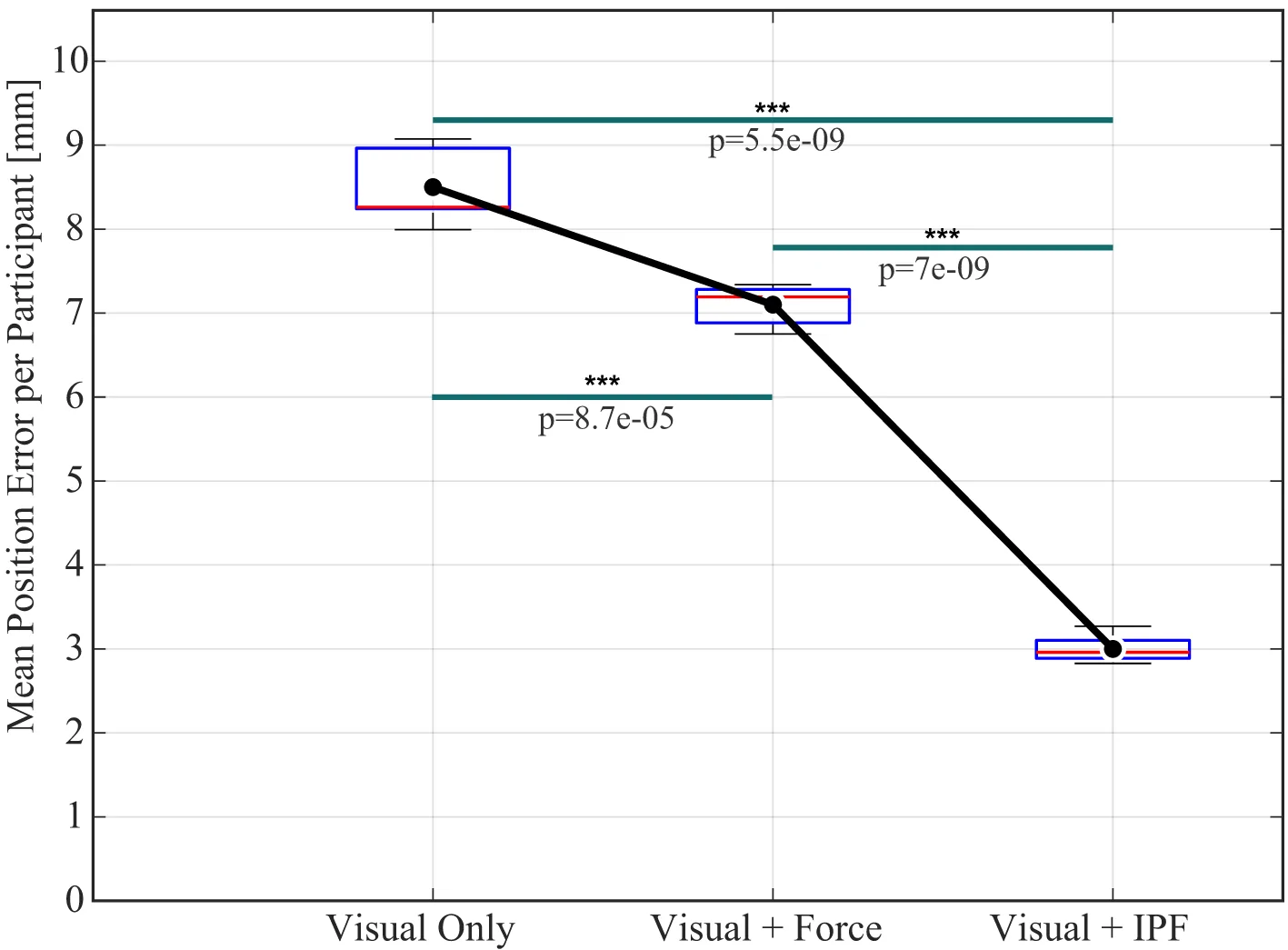
Participant-wise grasp-control performance under the three feedback conditions (Visual-only, Visual + Force, and Visual + IPF; *n* = 8). Boxplots summarize the distribution of participant-wise mean position errors, where the central line denotes the median, the box indicates the interquartile range, and the whiskers represent the data spread. Individual markers correspond to the mean error of each participant. Horizontal bars indicate the planned pairwise statistical comparisons between feedback conditions after Holm's sequential correction. The symbol ^***^indicates statistical significance at *p* < 0.001.

#### 3.1.1 Participant-wise performance across feedback conditions

When averaged across all trials and participants (*n* = 8), the *visual-only* condition resulted in the largest positional error, with a mean of approximately 8.5mm (SD ≈ 1.2). The addition of force feedback yielded a moderate improvement, reducing the mean error to 7.1mm (SD ≈ 1.0). The most notable change was observed in the *visual + IPF* condition, where error values dropped markedly to 3.0mm (SD ≈ 0.8).

Statistical comparisons confirmed these performance differences. Following Holm's sequential correction for the three predefined pairwise comparisons, significant differences remained between Visual-only and Visual + Force (*p* = 8.7 × 10^−5^), Visual-only and Visual + IPF (*p* = 1.65 × 10^−8^), and Visual + Force and Visual + IPF (*p* = 1.40 × 10^−8^).

#### 3.1.2 Subsession-wise performance trends within each condition

Breaking the data down by subsessions (S1–S3) provided additional insights into short-term learning and adaptation. The subsession-wise performance distributions are presented in Figure 10.

**Figure 10:**
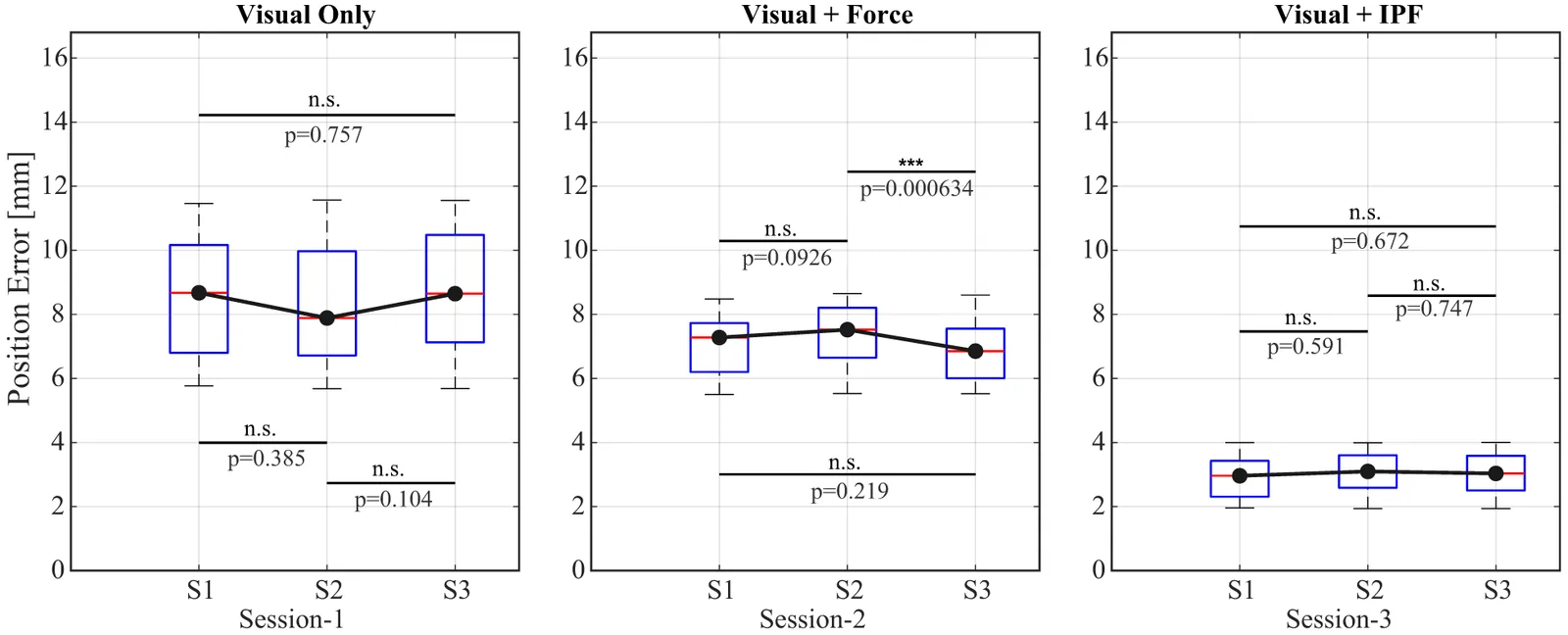
Subsession-wise distributions of position error under the three feedback modalities (Visual-only, Visual + Force, and Visual + IPF). Boxplots represent the distributions of participant-wise position errors for each subsession (S1–S3), where the central line denotes the median, the box indicates the interquartile range, and the whiskers represent the data spread. Statistical significance between subsessions is indicated where applicable. The symbol ^***^indicates statistical significance at *p* < 0.001.

**Visual-only condition:** Median errors remained relatively high across subsessions, ranging from 8.7mm in S1 to 8.4mm in S3, with no significant improvement after Holm's sequential correction (all adjusted *p* > 0.05).

**Visual + Force condition:** Errors showed a slight reduction, with median errors decreasing from 7.3mm to 6.9mm. Following Holm's sequential correction, only the comparison between S2 and S3 remained statistically significant (*p* = 0.0019).

**Visual + IPF condition:** Median errors remained consistently low across all three subsessions, varying only from approximately 3.2mm to 2.7mm. No statistically significant differences were observed between subsessions after Holm's sequential correction (all adjusted *p* > 0.05), indicating stable grasp-control performance throughout repeated interaction.

### 3.2 Experiment 2: Effect of frequency variation in IPF on grasping performance during repeated interaction

This experiment examined whether the stimulation frequency of IPF systematically influences grasp-control performance during repeated interaction. Three predetermined stimulation frequencies were evaluated: 5 Hz (low), 30 Hz (intermediate), and 60 Hz (high). All inferential analyses were performed at the participant level (*n* = 8). The selected frequencies also span physiologically relevant ranges of cutaneous mechanoreceptor sensitivity. Meissner (FA I) afferents are predominantly associated with frequencies of approximately 5–50 Hz, whereas Pacinian (FA II) afferents extend toward higher-frequency mechanical stimulation (approximately 40–500+ Hz); slowly adapting Merkel (SA I) and Ruffini (SA II) afferents are predominantly associated with lower-frequency mechanical stimulation (Li et al., 2017). Accordingly, 5 and 60 Hz provided low- and high-frequency conditions spanning distinct regions of cutaneous mechanoreceptive sensitivity, while 30 Hz was included as an intermediate comparison condition. These physiological considerations were combined with the practical bandwidth of the mechanical stimulator, for which 60 Hz represented the highest frequency that could be generated reliably under repeated stimulation.

A clear frequency-dependent reduction in position error was observed across the three stimulation conditions (Figure 11). Participant-level mean position error decreased from 2.47 ± 0.73mm at 5 Hz to 1.50 ± 0.23mm at 30 Hz and 1.01 ± 0.50mm at 60 Hz. The corresponding median errors were 2.37, 1.50, and 0.88 mm, respectively. Thus, both the mean and median values showed the same monotonic ordering, with the largest position errors occurring under low-frequency stimulation and the smallest errors under high-frequency stimulation.

**Figure 11:**
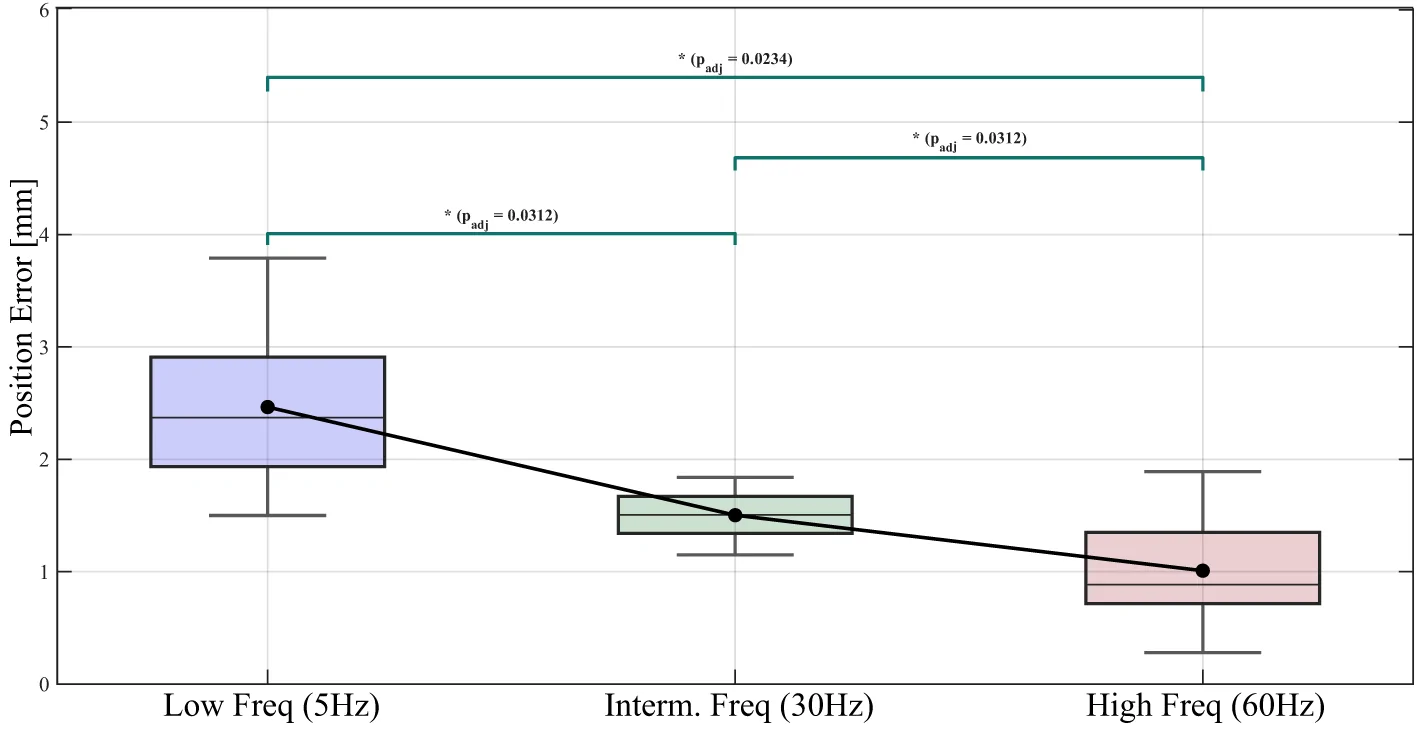
Participant-level comparison of grasp-control performance under three IPF stimulation frequencies (5, 30, and 60 Hz; *n* = 8). Boxplots show the distributions of participant-wise mean position errors, and black markers connected by lines indicate the corresponding group means. Statistical significance was first evaluated using a Friedman test, followed by predefined participant-level pairwise Wilcoxon signed-rank comparisons with Holm's sequential correction. Horizontal bars indicate significant pairwise comparisons, with the corresponding Holm-adjusted *p*-values (*p*_adj_). The symbol ^*^indicates statistical significance at *P*_adj_ < 0.05, where *P*_adj_ denotes the Holm-adjusted *p*-value.

A Friedman test confirmed a significant overall effect of stimulation frequency on position error [$\chi_{\left(2\right)}^{2}$ = 12.25, *p* = 0.0022]. Subsequent participant-level pairwise comparisons were performed using Wilcoxon signed-rank tests with Holm's sequential correction for the three predefined frequency comparisons. All pairwise differences remained statistically significant after correction: 5 vs. 30 Hz (*p*_adj_ = 0.0313), 5 vs. 60 Hz (*p*_adj_ = 0.0234), and 30 vs. 60 Hz (*p*_adj_ = 0.0313). The corresponding paired-sample effect sizes were *d*_*z*_ = 1.52, 2.29, and 1.44, respectively. The largest effect was therefore observed between the two extreme stimulation frequencies (5 and 60 Hz), consistent with the progressive reduction in position error observed across the frequency range. Collectively, these results indicate that stimulation frequency had a systematic influence on grasp-control accuracy within the frequency range evaluated in this study.

To further examine whether this frequency-dependent performance pattern was maintained across repeated experimental sessions, participant-level position errors were analyzed separately for Session 1 and Session 2 (Figure 12). Within-frequency paired comparisons revealed significant session-related changes at 5 Hz (*p*_adj_ = 0.0234, *d*_*z*_ = 1.40), 30 Hz (*p*_adj_ = 0.0313, *d*_*z*_ = 1.28), and 60 Hz (*p*_adj_ = 0.0313, *d*_*z*_ = 1.11), following Holm's sequential correction. Inspection of the paired participant trajectories in Figure 12 shows that these session-related changes occurred alongside substantial inter-participant variability rather than as identical changes across all participants. Importantly, the overall frequency-dependent ordering of position error remained evident across the repeated measurements, with lower errors associated with the higher stimulation frequencies. Accordingly, the session-wise analysis indicates that repeated exposure influenced absolute performance levels, while the principal frequency-dependent pattern identified by the participant-level analysis was retained.

**Figure 12:**
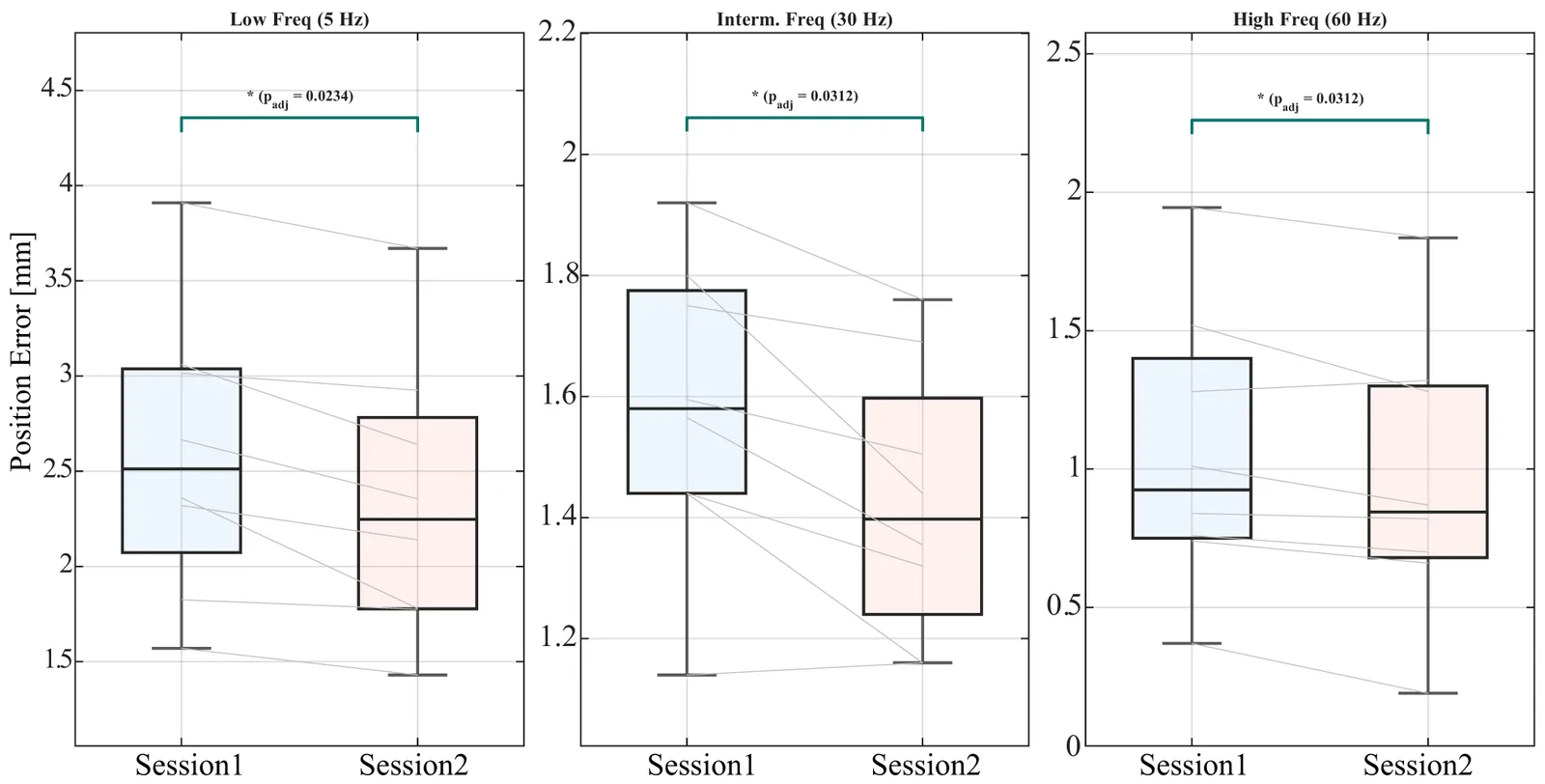
Session-wise participant-level position errors under the three IPF stimulation frequencies (5, 30, and 60 Hz; *n* = 8). Boxplots show the distributions of position errors for Session 1 and Session 2, while light-gray connecting lines represent paired observations from individual participants. Within each frequency condition, Session 1 and Session 2 were compared using participant-level Wilcoxon signed-rank tests with Holm's sequential correction across the three predefined comparisons. Horizontal bars report the corresponding Holm-adjusted *p*-values (*p*_adj_). The symbol ^*^indicates statistical significance at *p*_adj_ < 0.05, where *p*_adj_ denotes the Holm-adjusted *p*-value.

### 3.3 Experiment 3: Effects of error-driven frequency modulation on grasping performance

Experiment 3 investigated whether adapting stimulation frequency according to position error influences the temporal evolution of grasp-control performance during repeated interaction compared with constant high-frequency stimulation. Repeated tactile stimulation may reduce perceptual salience through short-term habituation; however, because perceptual habituation was not measured directly in the present experiment, changes in grasp-control performance were interpreted as behavioral responses to repeated stimulation rather than as direct evidence of habituation.

Two stimulation strategies were compared: fixed-frequency stimulation at 60 Hz and Progressive Frequency Modulation (PFM). During PFM, stimulation frequency was selected according to the error-dependent switching rule defined in Section 2.2, whereas the fixed-frequency condition maintained stimulation at 60 Hz throughout the experiment. Performance was evaluated across consecutive trials to determine whether the stimulation strategies differed in overall position-control accuracy and, importantly, whether their temporal performance profiles evolved differently during repeated interaction.

#### 3.3.1 Early-stage performance trends: PFM vs. fixed 60 Hz

Figure 13 illustrates the early-stage evolution of grasp-control performance across 15 consecutive trials. Position errors under PFM were descriptively lower than those under fixed 60-Hz stimulation across most of the trial sequence, although the magnitude of the difference varied across trials and the SEM envelopes overlapped during several portions of the sequence. Consistent with this temporal variability, the linear mixed-effects analysis revealed no significant overall main effect of stimulation condition [*F*_(1, 203)_ = 0.453, *p* = 0.502]. In contrast, a significant main effect of trial was observed [*F*_(14, 203)_ = 2.351, *p* = 0.0049], together with a significant trial-by-condition interaction [*F*_(14, 203)_ = 2.785, *p* < 0.001]. The significant interaction indicates that the temporal evolution of position error across the early trial sequence differed between PFM and fixed 60-Hz stimulation, despite the absence of a uniform overall difference between the two conditions during this initial interaction period. Trial-wise Cohen's *d* values are therefore presented only as descriptive measures of the magnitude of the between-condition differences and are not used for trial-specific statistical inference.

**Figure 13:**
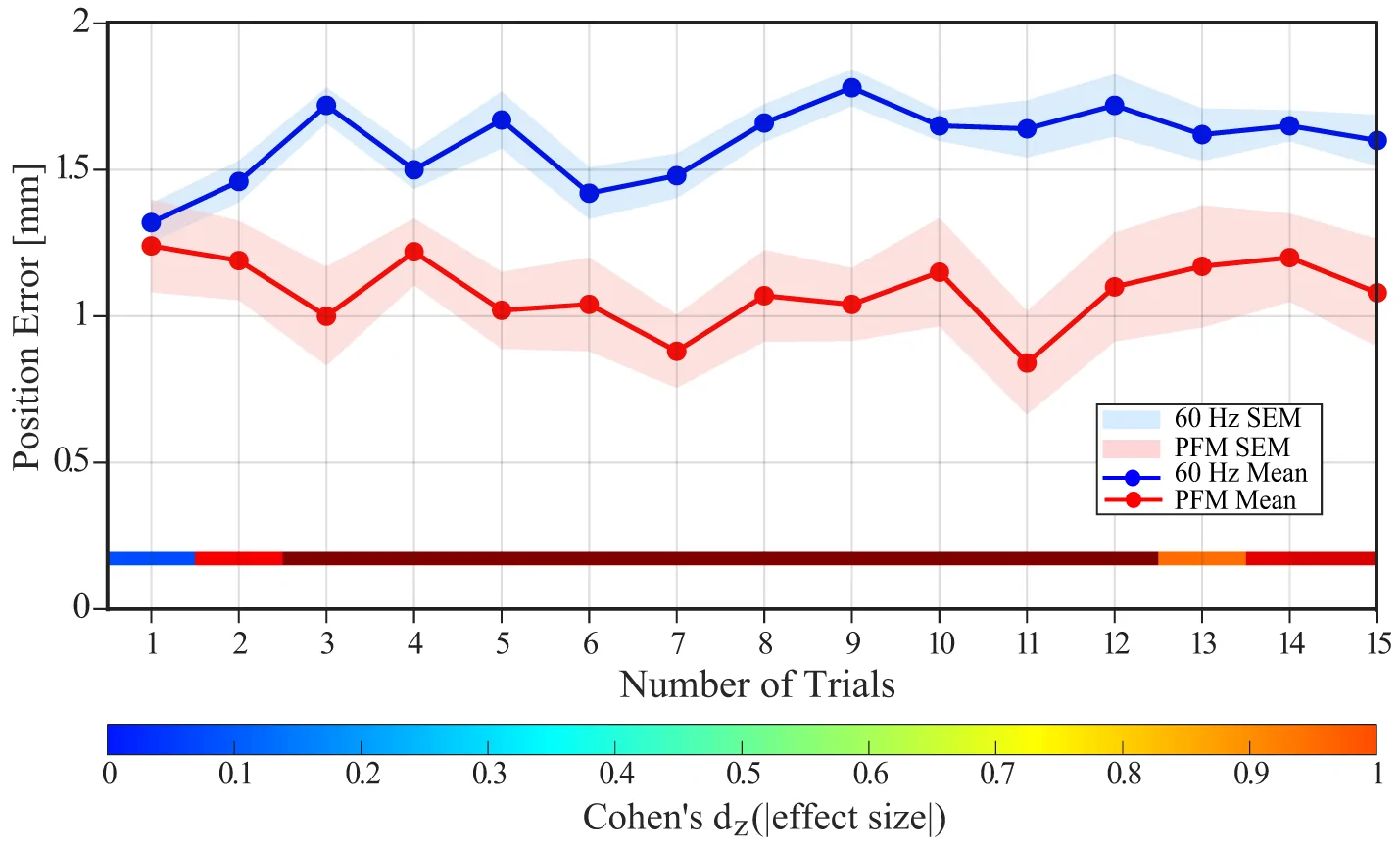
Early-stage evolution of grasp-control performance under Progressive Frequency Modulation (PFM) and fixed 60-Hz stimulation across 15 consecutive trials. Curves represent the mean position error across participants (*n* = 8), and shaded regions denote the standard error of the mean (SEM). For each trial, paired Cohen's *d*_*z*_ was calculated as the mean participant-wise difference between the fixed 60-Hz and PFM conditions divided by the standard deviation of the paired differences. The colored markers along the baseline represent the absolute magnitude of the trial-wise *d*_*z*_ values according to the color scale shown below the figure. These effect sizes are presented descriptively and were not used for trial-wise statistical inference. Statistical inference was performed using a linear mixed-effects model including stimulation condition, trial, and their interaction as fixed effects, with participant modeled as a random effect.

#### 3.3.2 Sustained performance during repeated interaction: PFM vs. fixed 60 Hz

Across the 35-trial repeated interaction, position error was generally lower under PFM than under fixed 60-Hz stimulation (Figure 14). At the participant level, the mean position error across the complete trial sequence was 1.680 ± 0.211 mm under fixed 60-Hz stimulation and 1.049 ± 0.284 mm under PFM (mean ± SD). The corresponding participant-level paired difference (60 Hz minus PFM) was 0.631 mm (95% CI: 0.243–1.019 mm), with a large paired effect size (*d*_*z*_ = 1.920).

**Figure 14:**
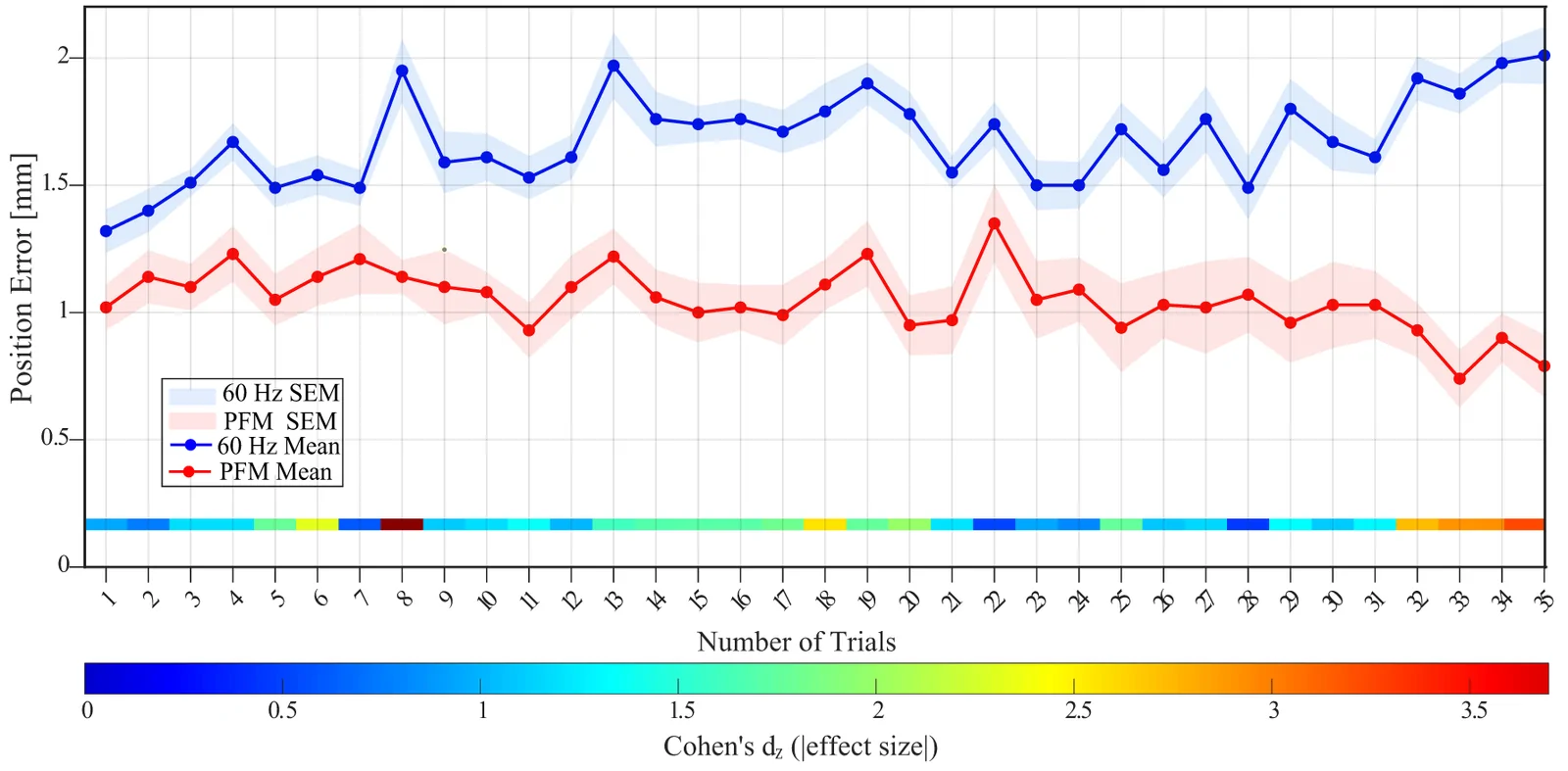
Sustained grasp-control performance under Progressive Frequency Modulation (PFM) and fixed 60-Hz stimulation across 35 consecutive trials (*n* = 8). Curves represent the mean position error across participants, and shaded regions denote the standard error of the mean (SEM). Statistical inference was performed using a linear mixed-effects model with stimulation condition, trial, and their interaction as fixed effects and participant as a random intercept, with trial treated as a categorical within-participant factor. For each trial, paired Cohen's *d*_*z*_ was calculated as the mean participant-wise difference between the fixed 60-Hz and PFM conditions divided by the standard deviation of the paired differences. The colored markers along the baseline represent the absolute magnitude of the trial-wise *d*_*z*_ values according to the color scale shown below the figure. These effect sizes are presented solely as descriptive measures; no trial-wise inferential tests were performed.

To account explicitly for the dependency among repeated observations from the same participant and to avoid multiple trial-by-trial significance tests, the complete 35-trial dataset was analyzed using a linear mixed-effects model. Stimulation condition, trial, and the Condition × Trial interaction were included as fixed effects, participant was modeled as a random intercept, and trial was treated as a categorical within-participant factor. The model revealed a significant main effect of stimulation condition [*F*_(1, 483)_ = 5.726, *p* = 0.017], demonstrating an overall difference in position error between the two stimulation strategies. A significant main effect of trial was also observed [*F*_(34, 483)_ = 3.997, *p* < 0.001], indicating that grasp-control performance varied across the repeated trial sequence. Importantly, the Condition × Trial interaction was significant [*F*_(34, 483)_ = 3.583, *p* < 0.001], demonstrating that the temporal evolution of position error differed between PFM and fixed 60-Hz stimulation.

The significant interaction indicates that the difference between the two stimulation strategies was not uniform across the trial sequence, consistent with the varying separation between the trajectories shown in Figure 14. Trial-wise paired Cohen's *d*_*z*_ values are therefore presented only to characterize the magnitude of these between-condition differences across the trial sequence and were not used for trial-specific statistical inference. This distinction is important because the inferential conclusions are derived from the mixed-effects model applied to the complete repeated-measures dataset rather than from a series of independent trial-by-trial comparisons.

Taken together, the participant-level descriptive statistics, large paired effect size, and mixed-effects analysis consistently indicate lower position errors under PFM during prolonged repeated interaction. Moreover, the significant Condition × Trial interaction demonstrates that the two stimulation strategies exhibited different temporal performance profiles. Because perceptual habituation was not measured directly, this temporal difference should not be interpreted as direct evidence that PFM reduced habituation. Rather, the findings provide behavioral evidence that error-dependent frequency modulation maintained lower grasp-control errors than constant 60-Hz stimulation during prolonged repeated interaction.

### 3.4 Experiment 4: Influence of spatial stimulation configuration (single-site vs. sequential dual-site stimulation) on position control accuracy

The spatial organization of tactile stimulation may influence how sensory feedback is perceived and subsequently used for motor control. While the temporal and intensity characteristics of tactile stimulation have been widely investigated, the functional consequences of distributing feedback across multiple stimulation sites remain comparatively less explored in prosthetic feedback systems. Accordingly, this experiment compared single-site stimulation with sequential dual-site stimulation to examine whether spatial stimulation configuration affects position-control performance during repeated interaction. The analysis evaluated the overall effect of spatial configuration, changes in performance across consecutive trials, and whether the temporal evolution of position error differed between the two stimulation configurations.

As shown in Figure 15, sequential dual-site stimulation generally yielded lower mean position errors than single-site stimulation across the 20-trial sequence, although the magnitude of the difference varied between trials. When summarized at the participant level across all trials, mean position error was 1.221 ± 0.166 mm under single-site stimulation and 0.898 ± 0.284 mm under sequential dual-site stimulation (mean ± SD). The participant-level paired difference (single-site minus sequential dual-site) was 0.323 mm (95% CI: 0.047–0.600 mm), corresponding to a large paired effect size (*d*_*z*_ = 0.979).

**Figure 15:**
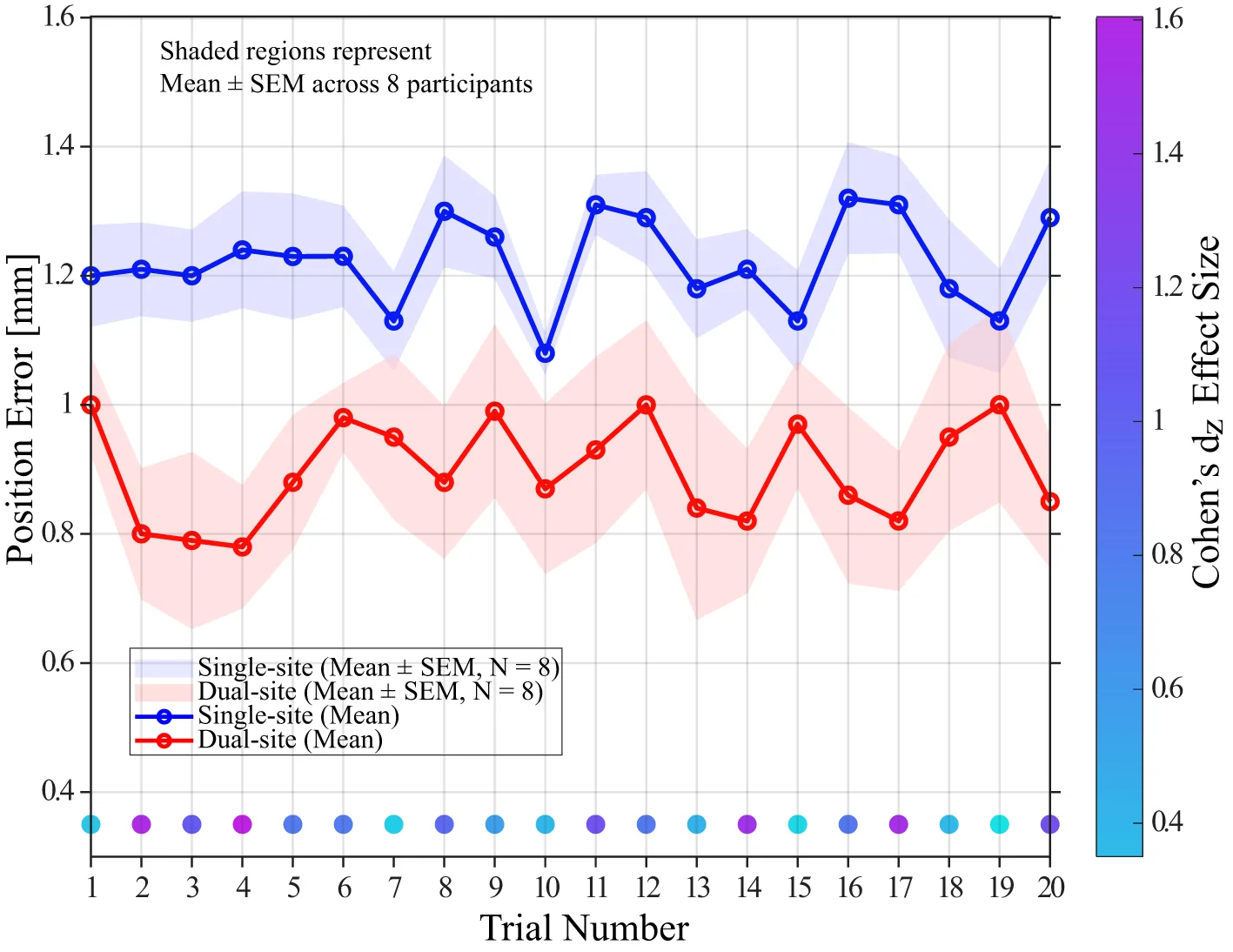
Position-control performance under single-site and sequential dual-site stimulation across 20 consecutive trials (*n* = 8). Curves represent the mean position error across participants, and shaded regions denote the standard error of the mean (SEM). Statistical inference was performed using a linear mixed-effects model with spatial configuration, trial, and their interaction as fixed effects and participant as a random intercept, with trial treated as a categorical within-participant factor. Trial-wise paired Cohen's *d*_*z*_ values are shown along the baseline solely as descriptive measures of effect magnitude; no trial-wise inferential tests were performed.

To account for the dependency among repeated observations from the same participant and to avoid multiple trial-by-trial significance tests, the complete 20-trial dataset was analyzed using a linear mixed-effects model. Spatial configuration, trial, and the Spatial Configuration × Trial interaction were included as fixed effects, participant was modeled as a random intercept, and trial was treated as a categorical within-participant factor. The omnibus analysis did not identify a statistically significant main effect of spatial configuration [*F*_(1, 273)_ = 2.723, *p* = 0.100]. The main effect of trial was also not significant [*F*_(19, 273)_ = 0.664, *p* = 0.853], indicating no systematic overall change in position error across the repeated trial sequence. Similarly, the Spatial Configuration × Trial interaction was not significant [*F*_(19, 273)_ = 0.869, *p* = 0.622], indicating that the temporal evolution of position error did not differ significantly between the two stimulation configurations.

Thus, although sequential dual-site stimulation was associated with a 0.323-mm lower participant-level mean position error and a large observed paired effect size (*d*_*z*_ = 0.979), the mixed-effects analysis did not provide sufficient evidence to establish a statistically significant overall effect of spatial configuration in the present sample. Importantly, the absence of significant Trial and Spatial Configuration × Trial effects indicates that the observed between-configuration difference did not arise from a systematic trial-dependent divergence of the two performance trajectories. Trial-wise Cohen's *d*_*z*_ values in Figure 15 are therefore provided only to characterize the magnitude of the observed paired differences and were not subjected to separate trial-wise hypothesis tests.

Taken together, these findings indicate a substantial participant-level effect magnitude in favor of sequential dual-site stimulation, but do not provide confirmatory statistical evidence of its superiority under the mixed-effects framework. The spatial-configuration effect should therefore be regarded as a promising behavioral tendency that warrants confirmation in a larger participant sample.

## 4 Discussion

This study investigated whether mechanonociceptive feedback can provide functionally useful information during boundary-constrained prosthetic-like grasping and how its effectiveness is influenced by stimulation frequency, temporal modulation, and spatial configuration. Across the experimental evaluations, a consistent overall pattern emerged: IPF was associated with lower grasp-position errors than the conventional feedback conditions tested in this study, while its behavioral effectiveness was further influenced by the temporal and spatial structure of stimulation. Importantly, these findings should be interpreted at the participant level and within the controlled experimental paradigm used here. With eight participants, the present experiments provide proof-of-concept evidence regarding the direction and consistency of the observed effects rather than population-level evidence of clinical efficacy.

The comparison of feedback modalities showed a graded performance pattern, with the largest position errors occurring under visual-only feedback, lower errors when force feedback was added, and the lowest errors under IPF. This progression is important because it indicates that the benefit of IPF cannot be attributed simply to the presence of an additional sensory channel. Rather, the type and perceptual salience of the supplementary information appear to influence how effectively participants detect and correct boundary violations during grasping. Mechanically evoked sharp or painful sensations are perceptually salient and can provide behaviorally relevant information about potentially excessive mechanical interaction (Greenspan and McGillis, 1991; Rolke et al., 2006). In the present task, however, IPF was not intended to reproduce biological pain or to induce an aversive state; it was used as a controlled mechanonociceptive cue indicating excessive displacement beyond the prescribed grasp boundary.

The subsession analysis provides additional information about how these modality-dependent differences evolved during repeated interaction. Visual-only performance remained comparatively poor across subsessions and did not show a significant improvement after correction for multiple comparisons, suggesting that repetition alone was insufficient to improve performance when boundary information was conveyed only visually. The visual-plus-force condition showed a modest reduction in error, indicating a more limited late-stage improvement. In contrast, the overall advantage associated with IPF was maintained across the repeated task structure. These results should not be interpreted as direct evidence of motor learning, because retention or transfer was not independently tested. Rather, they indicate that the availability and structure of sensory feedback affected performance throughout repeated interaction and that the lower errors observed with IPF were not restricted to the initial exposure period.

The frequency-characterization experiments further showed that the functional effectiveness of IPF depended on stimulation frequency. Within the frequencies evaluated in this study, high-frequency stimulation produced the lowest error levels, whereas intermediate- and low-frequency stimulation resulted in larger errors. This frequency dependence indicates that the same feedback modality can produce different behavioral outcomes depending on its temporal stimulation parameters. Such an effect is plausible because mechanically evoked cutaneous perception depends not only on stimulus magnitude but also on the temporal pattern through which sensory information is delivered. Nevertheless, the present findings should not be interpreted as evidence that increasing stimulation frequency monotonically improves performance. They identify a behavioral advantage for the higher-frequency condition within the tested parameter range only. Consequently, stimulation frequency should be regarded as an interface parameter requiring empirical optimization rather than as a universally scalable determinant of feedback effectiveness.

The comparison of fixed-frequency and progressively modulated stimulation provides further evidence that temporal structure is relevant during repeated feedback exposure. Progressive frequency modulation generally produced lower position errors than fixed-frequency stimulation, although the magnitude of the difference varied across the trial sequence. Repeated sensory stimulation is known to produce habituation and adaptation phenomena (Thompson, 2009; Barry, 2012; Milne et al., 1991); however, perceptual habituation was not directly measured in the present study. The behavioral results therefore cannot establish habituation as the mechanism underlying the difference between stimulation patterns. A more conservative interpretation is that progressive modulation preserved task-relevant feedback effectiveness more successfully than the fixed-frequency pattern under the tested conditions. Direct measurement of perceived intensity, detection thresholds, and perceptual adaptation during prolonged stimulation will be necessary to determine whether reduced habituation contributes to this effect.

Spatial stimulation configuration also affected performance. Sequential dual-site stimulation yielded lower overall position errors than repeated single-site stimulation, with participant-level mean errors of (0.898 ± 0.284) mm and (1.221 ± 0.166) mm, respectively. The separation between conditions was not uniform across the complete trial sequence, indicating that the advantage of dual-site stimulation should not be interpreted as a constant effect at every stage of interaction. Nevertheless, the descriptive participant-level difference suggests that distributing sequential feedback across two stimulation sites may improve the functional use of repeated mechanonociceptive cues. Several mechanisms could potentially contribute to this pattern. Spatial alternation may increase the distinctiveness of successive feedback events, provide a richer spatial representation, or reduce repeated stimulation of the same cutaneous location. Because the present experiment was not designed to distinguish among these possibilities, the result supports sequential multi-site stimulation as a promising interface strategy rather than establishing a particular perceptual mechanism.

Taken together, the present results indicate that the effectiveness of mechanonociceptive feedback depends not only on the feedback modality but also on its temporal and spatial encoding. For quantitative context, correct identification rates of 95.0% and 75.7% for four and seven prosthetic hand postures, respectively, have been reported using combined artificial somatosensory percepts in an individual with transradial amputation (Segil et al., 2020). Error-free identification of four prosthetic grip positions and an overall object-recognition success rate of 90.5% have also been achieved using combined mechanotactile and vibrotactile feedback (Sariyildiz et al., 2023). In closed-loop electrotactile tracking, normalized RMS tracking error increased from 9.4% at 100 Hz to 15.3% at 5 Hz, accompanied by an increase in response delay from 359.8 to 1,009 ms (Paredes et al., 2015). Although these values are not directly comparable with the millimeter-scale position errors measured in the present boundary-constrained grasping task because of differences in feedback modality, task, and outcome measure, they provide quantitative context for existing artificial tactile feedback approaches and demonstrate the dependence of feedback effectiveness on encoding parameters. The present findings extend this parameter-dependent design perspective to mechanonociceptive feedback.

An important limitation of the present study concerns ecological and clinical validity. The experiments were intentionally conducted in neurologically intact participants using a controlled prosthetic-like virtual grasping paradigm rather than in individuals with upper-limb amputation using a physical prosthesis. This design enabled the effects of feedback modality and stimulation parameters to be systematically isolated while reducing variability associated with amputation level, residual-limb condition, prosthetic experience, socket characteristics, and long-term sensory adaptation. At the same time, it does not reproduce the complete sensory, biomechanical, and functional conditions encountered during everyday prosthesis use. In particular, virtual boundary crossing and corrective finger motion cannot capture all factors involved in physical object interaction, including object compliance, friction, prosthetic mechanics, socket–limb interaction, and the consequences of excessive grasp force. Accordingly, the present findings should be interpreted as controlled proof-of-concept evidence for mechanonociceptive feedback and its parameter dependence, rather than as direct evidence of clinical benefit in prosthesis users.

Clinical translation also requires careful consideration of the tolerability and calibration of mechanonociceptive stimulation. Perceptual ranges established in neurologically intact tissue cannot be assumed to transfer directly to a residual limb, where mechanical and nociceptive sensitivity may vary substantially among individuals. Therefore, the stimulation amplitudes identified in the present healthy cohort should not be directly prescribed for amputee users. Translation of the proposed approach will require participant-specific calibration at the intended residual-limb stimulation site to determine perceptual and discomfort thresholds and establish a clearly tolerated operating range.

Future work will consequently proceed from controlled characterization toward amputee-specific functional validation. The proposed feedback will first be individually calibrated at the residual limb and subsequently evaluated with physical prosthetic devices during manipulation of objects with different mechanical properties and task demands. Future studies with individuals with upper-limb amputation should complement position-based performance measures with task-level outcomes, such as object-handling success and task-completion time, together with perceptual and user-centered measures including stimulus intensity, discomfort, cognitive workload, embodiment, and usability. Longer-duration experiments will be necessary to determine whether the advantages associated with progressive modulation and sequential multi-site stimulation persist beyond the relatively short interaction periods evaluated here. Direct psychophysical measurements should also accompany behavioral outcomes to determine whether changes in perceived intensity or habituation explain the observed temporal effects.

Taken together, the present study demonstrates that mechanonociceptive feedback can provide behaviorally useful error information during controlled prosthetic-like grasping and, importantly, that its effectiveness is not determined by modality alone. These findings establish a systematic experimental basis for optimizing mechanonociceptive feedback and motivate its subsequent evaluation in individuals with upper-limb amputation under physically and clinically representative prosthesis-use conditions.

## 5 Conclusion

This study investigated IPF as a mechanonociceptive tactile feedback modality for boundary-constrained grasp control during repeated human-in-the-loop prosthetic-like interaction. Within the controlled experimental paradigm, IPF was associated with lower position errors than both visual-only and visual-plus-force feedback, demonstrating that mechanonociceptive stimulation can provide behaviorally useful error-related information during grasp execution.

Beyond feedback modality, the results showed that performance was also influenced by how mechanonociceptive stimulation was delivered. Within the tested frequency range, high-frequency stimulation produced the lowest position errors, while progressive frequency modulation generally maintained lower error levels than fixed-frequency stimulation across repeated interaction. Sequential dual-site stimulation also yielded lower overall participant-level position errors than repeated single-site stimulation. Together, these findings indicate that the functional effectiveness of mechanonociceptive feedback depends not only on the availability of the sensory cue, but also on its temporal and spatial organization.

The present findings should be interpreted as proof-of-concept evidence obtained in neurologically intact participants using a virtual prosthetic-like grasping paradigm rather than as evidence of clinical efficacy in prosthesis users. In particular, the stimulation parameters and perceptual ranges established in intact tissue cannot be directly transferred to the residual limb, where sensory and nociceptive sensitivity may differ substantially across individuals. Translation of the proposed approach will therefore require participant-specific calibration and validation in individuals with upper-limb amputation using physical prostheses and functional object-manipulation tasks. Future studies should additionally determine whether the observed advantages of progressive temporal modulation and sequential multi-site stimulation persist during prolonged use and whether they translate into improvements in functional performance, tolerability, and user experience. These findings provide a basis for the systematic optimization and subsequent clinical evaluation of mechanonociceptive feedback as a sensory feedback strategy for prosthetic control.

## Data Availability

The raw data supporting the conclusions of this article will be made available by the authors, without undue reservation.

## References

[B1] Al-Ajam Y. Woollard A. Kang N. (2022). Advances in upper limb loss rehabilitation: the role of targeted muscle reinnervation and regenerative peripheral nerve interfaces. Plast. Aesthet. Res. 9:63. doi: 10.20517/2347-9264.2022.24

[B2] Antfolk C. D'Alonzo M. Rosén B. Lundborg G. Sebelius F. Cipriani C. (2013). Sensory feedback in upper limb prosthetics. Expert Rev. Med. Devices 10, 45–54. doi: 10.1586/erd.12.68 23278223

[B3] Asran H. Alfaris Z. Hocaoglu E. (2025). “Preliminary evaluation of the passive rehabilitation phase of the REACT system for tendon therapy,” in 2025 medical technologies congress (TIPTEKNO) (Gazi Magusa: IEEE), 1–4. doi: 10.1109/TIPTEKNO68206.2025.11270099

[B4] Barry R. J. (2012). “Habituation and sensitization,” in Encyclopedia of the Sciences of Learning, ed. N. M. Seel (Boston, MA: Springer), 1413–1414. doi: 10.1007/978-1-4419-1428-6_1053

[B5] Bates T. J. Fergason J. R. Pierrie S. N. (2020). Technological advances in prosthesis design and rehabilitation following upper extremity limb loss. Curr. Rev. Musculoskelet. Med. 13, 485–493. doi: 10.1007/s12178-020-09656-6 32488625 PMC7340716

[B6] Battezzato A. (2014). Towards an underactuated finger exoskeleton: an optimization process of a two-phalange device based on kinetostatic analysis. Mech. Mach. Theory 78, 116–130. doi: 10.1016/j.mechmachtheory.2014.03.007

[B7] Biddiss E. Chau T. (2007). Upper-limb prosthetics: critical factors in device abandonment. Am. J. Phys. Med. Rehab. 86, 977–987. doi: 10.1097/PHM.0b013e3181587f6c 18090439

[B8] D'Anna E. Valle G. Mazzoni A. Strauss I. Iberite F. Patton J. . (2019). A closed-loop hand prosthesis with simultaneous intraneural tactile and position feedback. Sci. Robot. 4:eaau8892. doi: 10.1126/scirobotics.aau8892 33137741

[B9] del Valle J. Navarro X. (2013). Interfaces with the peripheral nerve for the control of neuroprostheses. Int. Rev. Neurobiol. 109, 63–83. doi: 10.1016/B978-0-12-420045-6.00002-X 24093606

[B10] Dietrich C. Walter-Walsh K. Preissler S. Hofmann G. O. Witte O. W. Miltner W. H. R. . (2012). Sensory feedback prosthesis reduces phantom limb pain: proof of a principle. Neurosci. Lett. 507, 97–100. doi: 10.1016/j.neulet.2011.10.068 22085692

[B11] Fifer M. S. McMullen D. P. Osborn L. E. Thomas T. M. Christie B. Nickl R. W. . (2022). Intracortical somatosensory stimulation to elicit fingertip sensations in an individual with spinal cord injury. Neurology 98, e679–e687. doi: 10.1212/WNL.0000000000013173 34880087 PMC8865889

[B12] Fisher L. E. Gaunt R. A. Huang H. (2023). Sensory restoration for improved motor control of prostheses. Curr. Opin. Biomed. Eng. 28:100498. doi: 10.1016/j.cobme.2023.100498 37860289 PMC10583965

[B13] George J. A. Kluger D. T. Davis T. S. Wendelken S. M. Okorokova E. V. He Q. . (2019). Biomimetic sensory feedback through peripheral nerve stimulation improves dexterous use of a bionic hand. Sci. Robot. 4:eaax2352. doi: 10.1126/scirobotics.aax2352 33137773

[B14] Gescheider G. A. (1997). Psychophysics: The Fundamentals, 3rd Edn. Mahwah, NJ: Lawrence Erlbaum Associates.

[B15] Graczyk E. Hutchison B. Valle G. Bjanes D. Gates D. Raspopovic S. . (2024). Clinical applications and future translation of somatosensory neuroprostheses. J. Neurosci. 44:e1237242024. doi: 10.1523/JNEUROSCI.1237-24.2024 39358021 PMC11450537

[B16] Graczyk E. L. Schiefer M. A. Saal H. P. Delhaye B. P. Bensmaia S. J. Tyler D. J. (2016). The neural basis of perceived intensity in natural and artificial touch. Sci. Transl. Med. 8:362ra142. doi: 10.1126/scitranslmed.aaf5187 27797958 PMC5713478

[B17] Greenspan J. D. McGillis S. L. (1991). Stimulus features relevant to the perception of sharpness and mechanically evoked cutaneous pain. Somatosens. Motor Res. 8, 137–147. doi: 10.3109/08990229109144738 1887724

[B18] Hocaoglu E. (2019). “WeFiTS: wearable fingertip tactile sensor,” in Wearable Robotics: Challenges and Trends, Vol. 22, Biosyst. Biorobot, eds. M. Carrozza, S. Micera, and J. Pons (Cham: Springer International Publishing), 28–32. doi: 10.1007/978-3-030-01887-0_6

[B19] Hocaoglu E. (2023). Wearable thimble-like device for the objective follow-up and therapy of multiple sclerosis. Balkan J. Electr. Comput. Eng. 11, 249–256. doi: 10.17694/bajece.1295322

[B20] Hocaoglu E. Patoglu V. (2022a). Design, implementation, and evaluation of a variable stiffness transradial hand prosthesis. Front. Neurorobot. 16:789210. doi: 10.3389/fnbot.2022.789210 35360829 PMC8960622

[B21] Hocaoglu E. Patoglu V. (2022b). sEMG-based natural control interface for a variable stiffness transradial hand prosthesis. Front. Neurorobot. 16:789341. doi: 10.3389/fnbot.2022.789341 35360833 PMC8963738

[B22] Iqbal J. Tsagarakis N. G. Caldwell D. G. (2014). Human hand compatible underactuated exoskeleton robotic system. Electron. Lett. 50, 494–496. doi: 10.1049/el.2014.0508

[B23] Isaković M. Malešević J. Keller T. Kostić M. Štrbac M. (2019). Optimization of semiautomated calibration algorithm of multichannel electrotactile feedback for myoelectric hand prosthesis. Appl. Bionics Biomech. 2019:9298758. doi: 10.1155/2019/9298758 31001360 PMC6437744

[B24] Karakuş İ. Güçlü B. (2020). Psychophysical principles of discrete event-driven vibrotactile feedback for prostheses. Somatosens. Motor Res. 37, 186–203. doi: 10.1080/08990220.2020.1769055 32448043

[B25] Li K. Fang Y. Zhou Y. Liu H. (2017). Non-invasive stimulation-based tactile sensation for upper-extremity prosthesis: a review. IEEE Sens. J. 17, 2625–2635. doi: 10.1109/JSEN.2017.2674965

[B26] Milne R. J. Kay N. E. Irwin R. J. (1991). Habituation to repeated painful and non-painful cutaneous stimuli: a quantitative psychophysical study. Exp. Brain Res. 87, 438–444. doi: 10.1007/BF00231861 1769394

[B27] Mobius Bionics (2026). LUKE Arm. Available online at: https://www.mobiusbionics.com/luke-arm/ (Accessed August 08, 2026).

[B28] Ng K. K. W. Snow I. N. Birznieks I. Vickery R. M. (2021). Burst gap code predictions for tactile frequency are valid across the range of perceived frequencies attributed to two distinct tactile channels. J. Neurophysiol. 125, 687–692. doi: 10.1152/jn.00662.2020 33439792

[B29] Nghiem B. T. Sando I. C. Gillespie R. B. McLaughlin B. L. Gerling G. J. Langhals N. B. . (2015). Providing a sense of touch to prosthetic hands. Plast. Reconstr. Surg. 135, 1652–1663. doi: 10.1097/PRS.0000000000001289 26017599

[B30] Ortiz-Catalan M. (2018). The stochastic entanglement and phantom motor execution hypotheses: a theoretical framework for the origin and treatment of phantom limb pain. Front. Neurol. 9:748. doi: 10.3389/fneur.2018.00748 30237784 PMC6135916

[B31] Osborn L. E. Dragomir A. Betthauser J. L. Hunt C. L. Nguyen H. H. Kaliki R. R. . (2018). Prosthesis with neuromorphic multilayered e-dermis perceives touch and pain. Sci. Robot. 3:eaat3818. doi: 10.1126/scirobotics.aat3818 32123782 PMC7051004

[B32] Össur (2026). I-Limb Quantum. Available online at: https://www.ossur.com/en/prosthetics/arms/i-limb-quantum (Accessed August 08, 2026).

[B33] Page D. M. George J. A. Kluger D. T. Duncan C. Wendelken S. Davis T. . (2018). Motor control and sensory feedback enhance prosthesis embodiment and reduce phantom pain after long-term hand amputation. Front. Hum. Neurosci. 12:352. doi: 10.3389/fnhum.2018.00352 30319374 PMC6166773

[B34] Paredes L. P. Dosen S. Rattay F. Graimann B. Farina D. (2015). The impact of the stimulation frequency on closed-loop control with electrotactile feedback. J. Neuroeng. Rehabil. 12, 1–16. doi: 10.1186/s12984-015-0022-8 25889752 PMC4403675

[B35] Paskett M. D. Olsen N. R. George J. A. Kluger D. T. Brinton M. R. Davis T. S. . (2019). A modular transradial bypass socket for surface myoelectric prosthetic control in non-amputees. IEEE Trans. Neural Syst. Rehabil. Eng. 27, 2070–2076. doi: 10.1109/TNSRE.2019.2941109 31536008

[B36] Raspopovic S. Capogrosso M. Petrini F. M. Bonizzato M. Rigosa J. Di Pino G. . (2014). Restoring natural sensory feedback in real-time bidirectional hand prostheses. Sci. Transl. Med. 6:222ra19. doi: 10.1126/scitranslmed.3006820 24500407

[B37] Risso G. Valle G. (2022). Multisensory integration in bionics: relevance and perspectives. Curr. Phys. Med. Rehabil. Rep. 10, 123–130. doi: 10.1007/s40141-022-00350-x

[B38] Rolke R. Baron R. Maier C. Tölle T. R. Treede R.-D. Beyer A. . (2006). Quantitative sensory testing in the german research network on neuropathic pain (DFNS): standardized protocol and reference values. Pain 123, 231–243. doi: 10.1016/j.pain.2006.01.041 16697110

[B39] Sariyildiz E. Hanss F. Zhou H. Sreenivasa M. Armitage L. Mutlu R. . (2023). Experimental evaluation of a hybrid sensory feedback system for haptic and kinaesthetic perception in hand prostheses. Sensors 23:8492. doi: 10.3390/s23208492 37896585 PMC10611249

[B40] Segil J. L. Cuberovic I. Graczyk E. L. ff. Weir R. F. Tyler D. J. (2020). Combination of simultaneous artificial sensory percepts to identify prosthetic hand postures: a case study. Sci. Rep. 10:6576. doi: 10.1038/s41598-020-62970-4 32313060 PMC7171192

[B41] Shehata A. W. Rehani M. Jassat Z. E. Hebert J. S. (2020). Mechanotactile sensory feedback improves embodiment of a prosthetic hand during active use. Front. Neurosci. 14:263. doi: 10.3389/fnins.2020.00263 32273838 PMC7113400

[B42] Simon A. M. Lock B. A. Stubblefield K. A. (2012). Patient training for functional use of pattern recognition-controlled prostheses. J. Prosthet. Orthot. 24, 56–64. doi: 10.1097/JPO.0b013e3182515437 22563231 PMC3339840

[B43] Simon A. M. Turner K. L. Miller L. A. Dumanian G. A. Potter B. K. Beachler M. D. . (2023). Myoelectric prosthesis hand grasp control following targeted muscle reinnervation in individuals with transradial amputation. PLoS ONE 18:e0280210. doi: 10.1371/journal.pone.0280210 36701412 PMC9879512

[B44] Soghoyan G. Biktimirov A. Matvienko Y. Chekh I. Sintsov M. Lebedev M. A. (2023). Peripheral nerve stimulation enables somatosensory feedback while suppressing phantom limb pain in transradial amputees. Brain Stimul. 16, 756–758. doi: 10.1016/j.brs.2023.04.017 37100202

[B45] Steeper Group (2026). TASKA CX Hand. Available online at: https://www.steepergroup.com/prosthetics/upper-limb-prosthetics/hands/taska-cx/ (Accessed August 08, 2026).

[B46] Su S. Chai G. Meng J. Sheng X. Mouraux A. Zhu X. (2022). Towards optimizing the non-invasive sensory feedback interfaces in a neural prosthetic control. J. Neural Eng. 19:016028. doi: 10.1088/1741-2552/ac4e1b 35073525

[B47] Tan D. W. Schiefer M. A. Keith M. W. Anderson J. R. Tyler J. Tyler D. J. (2014). A neural interface provides long-term stable natural touch perception. Sci. Transl. Med. 6:257ra138. doi: 10.1126/scitranslmed.3008669 25298320 PMC5517305

[B48] TASKA Prosthetics (2024). How Does a Myoelectric Hand Work? Available online at: https://www.taskaprosthetics.com/about-us/news/myoelectric-hand/ (Accessed August 08, 2026).

[B49] Thompson R. F. (2009). Habituation: a history. Neurobiol. Learn. Mem. 92, 127–134. doi: 10.1016/j.nlm.2008.07.011 18703156 PMC2714193

[B50] Vincent Systems (2026). VINCENT Hand. Available online at: https://www.vincentsystems.de/en (Accessed August 08, 2026).

[B51] Wendelken S. Page D. M. Davis T. Wark H. A. C. Kluger D. T. Duncan C. . (2017). Restoration of motor control and proprioceptive and cutaneous sensation in humans with prior upper-limb amputation via multiple utah slanted electrode arrays (useas) implanted in residual peripheral arm nerves. J. Neuroeng. Rehabil. 14:121. doi: 10.1186/s12984-017-0320-4 29178940 PMC5702130

[B52] Xie A. Li C. Chou C.-H. Li T. Dai C. Lan N. (2024). A hybrid sensory feedback system for thermal nociceptive warning and protection in prosthetic hand. Front. Neurosci. 18:1351348. doi: 10.3389/fnins.2024.1351348 38650624 PMC11033464

